# Impact of a Text Messaging Intervention as an In-Between Support to Diabetes Group Visits in Federally Qualified Health Centers: Cluster Randomized Controlled Study

**DOI:** 10.2196/55473

**Published:** 2024-11-28

**Authors:** Allie Z Yan, Erin M Staab, Daisy Nuñez, Mengqi Zhu, Wen Wan, Cynthia T Schaefer, Amanda Campbell, Michael T Quinn, Arshiya A Baig

**Affiliations:** 1 Pritzker School of Medicine, University of Chicago, Chicago, IL, United States; 2 Department of Medicine, University of Chicago, Chicago, IL, United States; 3 Midwest Clinicians’ Network, East Lansing, MI, United States

**Keywords:** diabetes, diabetes mellitus, type 2 diabetes, mHealth, mobile health, digital health, digital technology, digital intervention, text messaging intervention, text message, text messaging, texting, federally qualified health center, FQHC, mobile phone

## Abstract

**Background:**

In the United States, 1 in 11 people receive primary care from a federally qualified health center (FQHC). Text messaging interventions (TMIs) are accessible ways to deliver health information, engage patients, and improve health outcomes in the health center setting.

**Objective:**

We aimed to evaluate the impact of a TMI implemented with a group visit (GV) intervention among patients with type 2 diabetes mellitus (T2DM) at FQHCs on patient-reported outcomes and clinical outcomes based on patient TMI engagement.

**Methods:**

A TMI was implemented for 11 health centers participating in a cluster randomized study of diabetes GVs in Midwestern FQHCs targeting adults with T2DM. FQHC patients participated in 6 monthly GVs either in person or online and a concurrent 25-week TMI. Outcome measures included clinical markers such as glycated hemoglobin A_1c _and patient-reported diabetes distress, diabetes self-care, diabetes self-efficacy, diabetes care knowledge, diabetes quality of life, diabetes social support, and TMI use and satisfaction. TMI response rate was calculated as responses to an SMS text message requesting a response divided by total messages requesting a response sent. Patients were grouped as high responders if their response rate was greater than or equal to the median response rate and low responders if their response rate was below the median. We conducted linear mixed models to compare high and low responders and within a group, adjusting for age, gender, GV attendance, and depression/anxiety at baseline.

**Results:**

In total, 101 of 124 GV patients (81.5%) enrolled in the TMI. The average age of the population in the TMI was 53 years. Of the 101 respondents, 61 (60%) were racial or ethnic minorities, while 42 of 82 respondents (51%) had a high school diploma/General Education Development or less, and 56 of 80 respondents (71%) reported an annual income less than US $30,000. In addition, 70 of 81 respondents (86%) owned a smartphone and 74 of 80 respondents (93%) had an unlimited texting plan. The median response rate was 41% and the mean response rate was 41.6%. Adjusted models showed significantly improved diabetes knowledge (*P*<.001), foot care (*P*<.001), and exercise (*P*=.002) in high responders (n=34) compared to low responders (n=23) at 6 months. No group difference was found in glycated hemoglobin A_1c_. Within high responders, diabetes distress (*P*=.001), social support (*P*<.001), quality of life (*P*<.001), diabetes knowledge (*P*<.001), foot care (*P*<.001), and diet (*P*=.003) improved from baseline to 6 months. Low responders only improved in diabetes quality of life (*P*=.003) from baseline to 6 months.

**Conclusions:**

In a FQHC safety net population participating in a combined TMI and GV intervention, our study showed improved diabetes distress, social support, knowledge, self-care, self-efficacy, and quality of life among patients highly engaged in the SMS text messaging program.

## Introduction

In the United States, 1 in 11 people receive primary care from a federally qualified health center (FQHC). FQHCs represent a population disproportionately comprised of people with lower socioeconomic status, racial and ethnic minorities, and those with a higher disease burden of uncontrolled diabetes [1]. Text messaging interventions (TMIs) have been identified as a promising method to improve clinical outcomes and health behaviors among patients with diabetes [2-8]. Many studies have found that TMIs have the potential to improve patient understanding of diabetes, self-efficacy, self-management behaviors, and clinical outcomes such as glycated hemoglobin A_1c_ (HbA_1c_) among adults with diabetes. TMIs have been understudied in vulnerable populations and FQHC patients, which share a disproportionate burden of diabetes [3,6,9]. A literature review conducted between January 2012 and February 2019 identified only 9 original articles studying T2DM TMIs in US adults that provided some participant demographics on race, income, or education [9]. Of these articles, only 4 of 9 reported English-speaking status and 2 of 9 reported income or education [9]. Today, though cellphone ownership is similar across racial groups, education levels, and income levels, disparities still exist with smartphone ownership and home broadband access [10,11]. With the increasing accessibility and broad use of cellphones and SMS text messaging among vulnerable populations, the barriers to TMIs are continuously decreasing, yet research focusing on vulnerable groups remains sparse [9].

TMIs are one of the most widely used mobile health tools and can be used for a wide variety of purposes including medication reminders, provider-patient communication, patient education, patient motivation, and data collection [2]. However, to date, very few studies have evaluated the usage of TMIs integrated with other clinic- and community-based interventions such as group visits (GVs). One prior study combined an interactive and tailored TMI with monthly phone coaching found significant treatment effects on HbA_1c_ at 3 months and 6 months that were not sustained at later follow-up [12]. GVs are a model of care that combines individual medical care with group education and social support. GVs have been found to be powerful tools in addressing health care inequalities, especially for vulnerable patients with chronic conditions such as diabetes [13,14]. The application of TMIs in addition to other interventions may be an effective approach to improve outcomes and serve to continue care and contact between patients and providers between visits.

The study of mobile health interventions in vulnerable populations represents both an emerging field of research and opportunity to utilize a powerful tool to serve disadvantaged patient populations that are disproportionately affected by diabetes [6,9]. Combining a TMI with an FQHC GV intervention is a novel approach to addressing diabetes and diabetes disparities. In this study, a combined diabetes GV and TMI was implemented at 11 Midwestern FQHCs with a diverse population of patients with diabetes with suboptimal glycemic control randomized into either a standard in-person cohort or an online GV cohort. The aim of this study was to assess how engagement with an SMS text messaging program correlated with clinical and patient-reported outcomes when implemented in the setting of a concurrent GV program.

## Methods

A cluster randomized controlled study was implemented, where FQHC staff conducted a 6-month GV and TMI at their site or online.

### Ethical Considerations

The University of Chicago Institutional Review Board (IRB 17-1385) approved all study procedures. The study was registered at ClinicalTrials.gov (NCT03487692) on April 4, 2018. Patients enrolled study provided informed consent. Patient data was shared by clinics with the University of Chicago as limited datasets and were saved on password protected, secure servers. Clinics engaged in the study received a stipend for their participation and could use the funds to offset any costs.

### Health Center Recruitment

The research team sent messages through the Midwest Clinicians’ Network (MWCN) listserv, set up a webinar through the MWCN, advertised in the quarterly MWCN e-newsletter, and mailed a letter and brochures about the study to the directors of all MWCN member health centers. Health centers were randomized to either the 2018 in-person intervention arm or the 2020 delayed intervention arm. The 2020 cohort was eventually adapted to an online format due to the onset of the COVID-19 pandemic. Initially, 16 health centers were enrolled and 5 withdrew. Of the remaining 11 health centers, 6 health centers with 7 clinical sites were randomized to the intervention cohort, and 5 health centers with 6 clinical sites were randomized to the 2020 delayed intervention. Health centers represented 7 Midwestern states including Indiana, Minnesota, Iowa, Wisconsin, Nebraska, Missouri, and Illinois. Specific cities represented both rural and urban settings. Due to a technical error, patients from 1 enrolled health center were not enrolled in the SMS text messaging program.

### Patient Recruitment

Eligible patients had to be at least 18 years of age, English- or Spanish-speaking, have a diagnosis of T2DM, and a most recently documented HbA_1c_ result in the last 6 months equal to or greater than 8.0%. Patients must have attended at least 2 appointments at the FQHC within the past year, with at least 1 of them being from the past 6 months to ensure that enrolled patients were actively engaged at the FQHC for care. Patients must have owned a cellphone with SMS text messaging capabilities and have had the ability to send and receive SMS text messages. Patients who were pregnant or planning to become pregnant or who had an uncontrolled psychiatric problem, dementia, another cognitive impairment, hearing difficulties, or a severe physical disability that would have excluded them from participation or benefiting from a GV were excluded. FQHC staff invited patients from a randomly ordered list until 15 patients met the eligibility criteria and agreed to participate. For the online 2020 cohort, FQHCs enrolled up to 12 patients due to the online format. Trained community health center (CHC) staff obtained written informed consent from all intervention participants prior to enrollment. Patients who consented and enrolled in the GV program were then offered the opportunity to also enroll in the SMS text messaging program, which was to run concurrently with the GV program. A total of 124 patients were enrolled in the GV program. There were 75 patients enrolled in the 2018 in person cohort and 49 patients enrolled in the 2020 online cohort.

### GV Intervention

The in-person FQHCs were asked to implement 6 monthly GVs lasting 2-3 hours at their care sites based on a previous, successful pilot study [13]. Participants in the online cohort were asked to implement 6 monthly group video visits lasting 1-2 hours. Due to the COVID-19 pandemic, the 2020 cohort had video-based GVs. The intervention was designed based on a review of the literature and input from staff and providers with experience implementing GVs in health center settings. The GVs contained several core components: (1) an individual medical visit with a provider, (2) group diabetes education, (3) group social support, and (4) goal setting.

Both in-person and online GVs included diabetes education led by a staff member or a guest speaker and a facilitator-led discussion to encourage peer support and goal setting. Due to the diversity of the CHCs and patients served, CHCs were able to use their own diabetes education curricula or curriculum resources provided during the training. The CHC staff and providers were encouraged to provide medication refills, provide referrals, order vaccinations and laboratory testing, and complete other process of care based on the American Diabetes Association Standards of Care during the GVs.

### TMI Implemented in the Study

**Figure 1.**
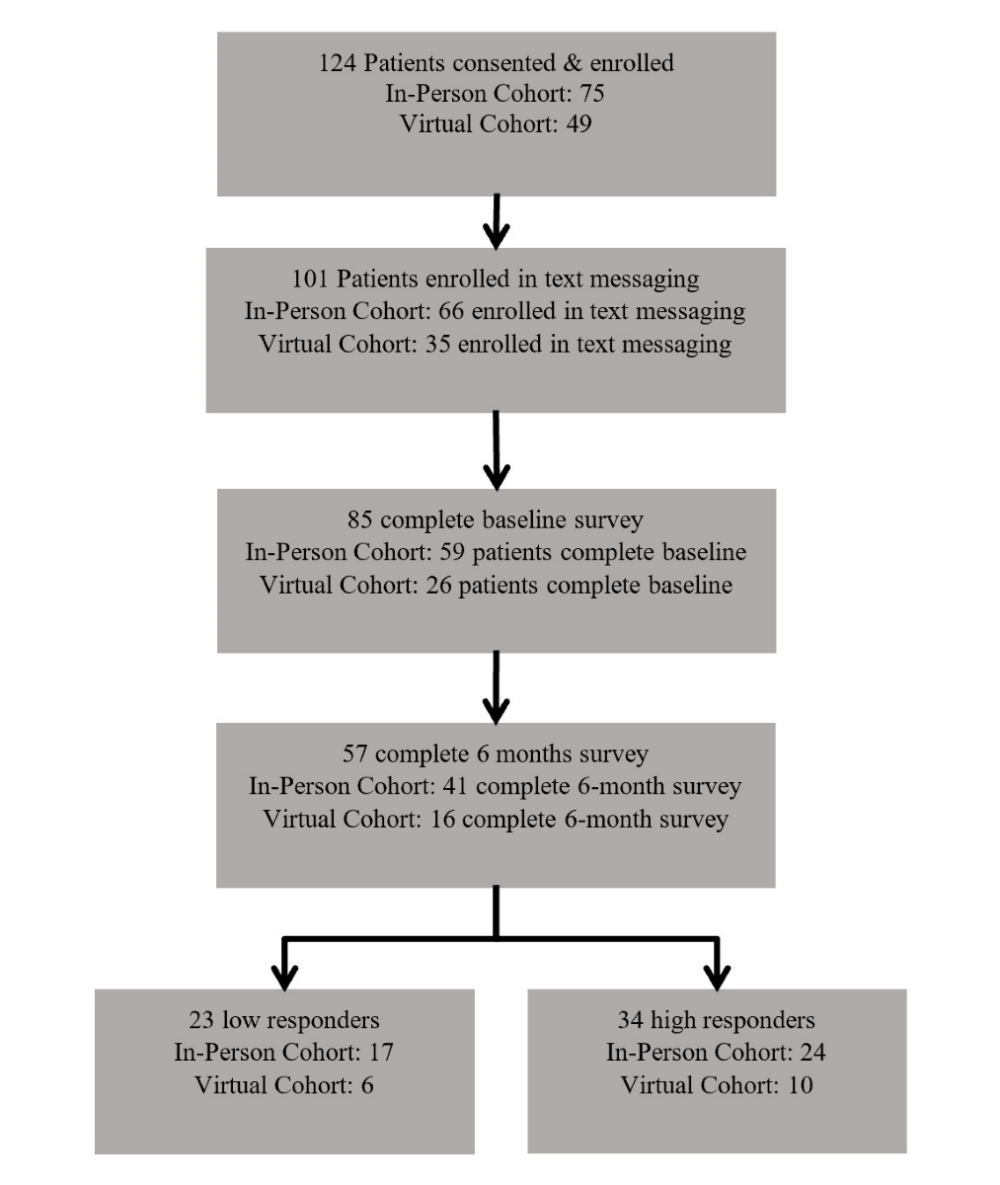
Enrollment and inclusion flowchart of in-person and online (virtual) cohorts.

CareMessage is a nonprofit health care patient engagement platform that serves clinical sites with underserved patient populations including FQHCs, free clinics, and other safety net care settings. It has an automated 25-week interactive T2DM SMS-based program focused on self-management and disease education based on national guidelines and supported by medical literature. Their program contains both unidirectional and bidirectional messages that ask for patient responses, including yes/no, true/false, and multiple-choice questions. Patients received 3‐5 text messages per week. Messages were available in both English and Spanish, written below a sixth-grade reading level, and culturally tailored for Black and Hispanic patients, with content designed to be practical and applicable for low-income patients. CareMessage provided training and worked with the FQHCs to implement the SMS text messaging platform within the clinic’s existing infrastructure and workflow. Of the 124 patients enrolled in the GV program, 104 patients enrolled in the SMS text messaging program, of which a total of 101 patients successfully received SMS text messages in the program. Full enrollment data are presented in Figure 1.

### Measures

Patients in both the in-person 2018 cohort and the online 2020 cohort completed surveys at baseline and 6 months, after the last GV. Patient surveys were administered by health center teams; there were multiple options for survey completion including on paper, by phone, or online. The patient surveys collected demographic data and data on patients’ access, comfort, and prior usage of technology such as texting. Surveys also assessed diabetes knowledge [15] via the Diabetes Knowledge Questionnaire (DKQ), diabetes self-management via the Summary of Diabetes Self-Care Activities (SDSCA) scale [16], SDSCA self-efficacy, diabetes-related distress via the Diabetes Distress Scale (DDS-2) [17], diabetes-related quality of life via the Diabetes Quality of Life (DQOL) Brief Clinical Inventory [18], and diabetes-related social support via the Diabetes Social Support Questionnaire (DSSQ) [18]. The SDSCA is divided into 5 sections: foot care, exercise, blood sugar testing, general diet, and specific diet. SDSCA self-efficacy is a survey we developed based on the SDSCA, which surveyed patients’ confidence in performing those activities. FQHC staff conducted chart reviews to collect patients’ clinical outcomes such as HbA_1c_. Chart data also contained some basic demographic data. Patient surveys also contained free-text portions regarding their experiences with CareMessage.

The CareMessage platform collected data on patient engagement, including the number of texts sent and received, response rates, and retention rates. FQHC teams sent these data reports with deidentified patient information to the University of Chicago team.

Postintervention, trained research team members from the University of Chicago team conducted 20- to 45-minute telephone interviews with FQHC staff. The interview questions were based on an interview guide designed to assess staff characteristics and involvement; barriers and facilitators to implementing and maintaining a diabetes GV intervention; characteristics of the GV intervention as implemented and adapted to their site; desire and ability to sustain the GV intervention; and patient and staff perceptions of the SMS text messaging program. Interviews were audio recorded then transcribed by a professional transcription company.

### Analysis

The baseline demographic information, diabetes-related health history, and patients’ access to, prior usage, and familiarity with SMS text messaging were described for each cohort separately and across both cohorts for patients that were enrolled in the TMI.

Response rate was calculated as the number of SMS text messages a patient replied to that were categorized as SMS text messages requesting a response, divided by the total number of SMS text messages that were categorized as ones requiring a response. To grade intervention engagement, patients were classified as high responders if their response rate was equal to or above the median response rate and low responders if their response rate was below the median response rate. Sensitivity analyses were also performed to evaluate response rate as a continuous variable. We defined the median response rate as the median response rate of all patients from both cohorts combined that enrolled in the SMS text messaging program.

All patient demographics and quantitative outcomes were summarized with descriptive statistics. Linear mixed models (for continuous variables) or generalized estimating equations (for categorical/binary variables) were used to adjust for within health center association. Each linear mixed model/generalized estimating equation was also used to examine TMI effects over time by testing the effects of the TMI and time and their interaction on each of the patient-reported outcomes and HbA_1c_. To consider any potential confounding effects, age, gender, number of GVs attended, and/or depression or anxiety at baseline were adjusted in these models. Through these models, we also conducted within-group comparison. Furthermore, we examined any cohort effect in each outcome and did not find any evidence to show significance. The interaction term between TMI responder status and GV attendance in a linear mixed model was not significant and was removed from the model. GV attendance was also not significant in the models and thus was not included. A 2-sided test with *P*<.05 was considered statistically significant.

To analyze patient free-text responses, 2 investigators used a modified template approach to text analysis to create an initial codebook [19]. Each member coded the free-text responses independently then met with the other to reach a consensus.

## Results

### Baseline Patient Characteristics

Table 1 describes the demographics and baseline characteristics of patients who enrolled in the SMS text messaging program (N=101). Their mean age was 53 (SD 12) years, 71% (72/101) were female, 60% (60/101) identified as racial or ethnic minorities, 20.7% (17/82) worked full-time, 51.2% (42/82) had completed high school, General Education Development, or less, and 73% (74/101) had public health insurance. Chart data were abstracted for every patient enrolled. Only some patients completed baseline surveys.

**Table 1.** Baseline characteristics of high and low responders (N=101).

Demographics	High responders		Low responders		All		*P* value
	Values	Participants, n	Values	Participants, n	Values	Participants, n	
**Age, mean (SD)**	51.1 (11.3)	50	55.0 (12.9)	51	53 (12.3)	101	.12
**Female, n (%)**	37 (74)	50	35 (69)	51	72 (71)	101	.62
**Race/ethnicity, n (%)**							.88
Hispanic or Latino	13 (26)	50	7 (14)	51	20 (20)	101	
Non-Hispanic Native American	3 (6)	50	5 (9.8)	51	8 (7.9)	101	
Non-Hispanic Asian	1 (2)	50	0	51	1 (1)	101	
Non-Hispanic Black	9 (18)	50	22 (43)	51	31 (31)	101	
Non-Hispanic White	23 (46)	50	17 (33)	51	40 (40)	101	
Other	1 (2)	50	0	51	1 (1)	101	
**Income, n (%)**							.94
Less than US $30,000	28 (68.3)	41	28 (71.8)	39	56 (70)	80	
US $30,000 to US $80,000	12 (29.3)	41	10 (25.6)	39	22 (27.5)	80	
Greater than US $80,000	1 (2.6)	41	1 (2.6)	39	2 (2.5)	80	
**Employment status, n (%)**							.35
Working full-time	12 (27.3)	44	5 (13.16)	38	17 (20.7)	82	
Working part-time	5 (11.4)	44	7 (18.4)	38	12 (14.6)	82	
Homemaker	5 (11.4)	44	3 (7.9)	38	8 (9.8)	82	
Retired	2 (4.6)	44	7 (18.4)	38	9 (11)	82	
Disabled	15 (34.1)	44	11 (29)	38	26 (31.7)	82	
Unemployed	4 (9.1)	44	5 (13.2)	38	9 (11)	82	
**Education, n (%)**							.26
High school, General Education Development, or less	20 (45.4)	44	22 (57.9)	38	42 (51.2)	82	
Some college or more	24 (54.6)	44	16 (42.1)	16	40 (48.8)	82	
**Insurance, n (%)**							.04
Private	9 (18)	50	2 (3.9)	51	11 (11)	101	
Public	31 (62)	50	43 (84)	51	74 (73)	101	
Self/uninsured	10 (20)	50	6 (12)	51	16 (16)	101	
**Preferred language, n (%)**							.97
English	42 (84)	50	47 (92)	51	89 (88)	101	
Spanish	8 (16)	50	4 (7.8)	51	12 (12)	101	
**Clinical characteristics**							
Age at diagnosis with diabetes, mean (SD)	39 (11)	45	41 (15)	38	39.9 (12.9)	83	.57
Duration of diabetes, mean (SD)	11.6 (7.8)	45	13.6 (11.1)	38	12.5 (9.5)	83	.33
Family history of diabetes, n (%)	41 (89.1)	46	34 (89.5)	38	75 (89.3)	84	.96
Glycated hemoglobin A_1c_, mean (SD)	9.02 (1.36)	50	9.60 (1.51)	51	9.31 (1.46)	101	.03
**Smoking, n (%)**							.85
Current smoker	10 (20)	50	11 (22)	51	21 (21)	101	
Former smoker	9 (18)	50	13 (25)	51	22 (22)	101	
Never smoker	31 (62)	50	27 (53)	51	58 (57)	101	
**Depression, n (%)**							
Patient Health Questionnaire positive (score ≥3)	9 (20)	45	16 (42.1)	38	25 (30.1)	83	.03
**Patient-reported medication use, n (%)**							
Uses insulin	31 (67.4)	46	32 (84.2)	38	63 (75)	84	.08
Uses other diabetes medications	40 (87)	46	33 (86.8)	38	73 (86.9)	84	.99
**Texting access, usage, and comfort**							
***How comfortable are you with text messaging on your phone? (1=very comfortable to 4=very uncomfortable), mean (SD)***	1.22 (0.60)	44	1.5 (0.81)	36	1.35 (0.71)	80	.09
Very comfortable, n (%)	37 (84.1)	44	24 (66.7)	36	61 (76.2)	80	
Somewhat comfortable, n (%)	5 (11.4)	44	7 (19.4)	36	12 (15)	80	
Somewhat uncomfortable, n (%)	1 (2.3)	44	4 (11.1)	36	5 (6.2)	80	
Very uncomfortable, n (%)	1 (2.3)	44	1 (2.8)	36	2 (2.5)	80	
**Sends text messages on their phone at least once per day, n (%)**	40 (90.9)	44	24 (64.9)	37	64 (79)	81	.004
**Receives text messages on their phone at least once per day, n (%)**	41 (93.2)	44	27 (73)	37	68 (84)	81	.01
**Owns a smartphone, n (%)**	40 (90.9)	44	30 (81.1)	37	70 (86.4)	81	.20
**Has an unlimited texting plan, n (%)**	43 (97.7)	44	31 (86.1)	36	74 (92.5)	80	.05
**Has received SMS text messages from a health care provider or clinic, n (%)**	29 (64.4)	45	18 (52.9)	34	47 (59.5)	79	.30
**Has sent SMS text messages to a health care provider or clinic, n (%)**	13 (28.9)	45	8 (23.5)	34	21 (26.6)	79	.69
**Has used a smartphone app to communicate with a health care provider or clinic, n (%)**	16 (35.6)	45	8 (24.2)	33	24 (30.8)	78	.29
**Has used a smartphone app to help them with self-care for their diabetes, n (%)**	9 (20.5)	44	2 (6.3)	32	14 (15.1)	76	.08

### Engagement With the TMI

Among those enrolled in the SMS text messaging program, the mean response rate was 41% (SD 37%). Of the 101 participants, 71 (70%) responded to at least 1 message. The mean number of SMS text messages sent was 89.7 (SD 23.8) and the mean number of SMS text messages requiring a response was 20.3 (SD 6.6). The mean number of days participants remained in the program was 157.5 (SD 42.2), ranging from 2 to 172 days. The median number of days in the program was 172. Among those that started the SMS text messaging program, 91 of 101 (90.1%) completed the program. Among those successfully enrolled in the SMS text messaging program, 51 patients were identified as low responders and 50 were identified as high responders. Of patients both enrolled in the SMS text messaging program and surveyed at baseline and 6 months successfully, 34 patients were classified as high responders and 23 were low responders. Sensitivity analyses were performed with response rate added as a continuous variable and the primary analysis results remained robust.

High and low responders differed in insurance status at baseline. Among high responders, 9 of 50 (18%) had private insurance, 31 of 50 (62%) had public insurance, and 10 of 50 (20%) were self-insured or uninsured. Among low responders, 2 of 51 (3.9%) had private insurance, 43 of 51 (84%) had public insurance, and 6 of 51 (12%) were self-insured or uninsured. High and low responders also differed significantly in GV attendance (*P*=.008). On average, high responders had an attendance of 3.58 (SD 1.99) GVs, while low responders had an average of 2.33 (2.03) GVs. High and low responders demonstrated differences in baseline HbA_1c_. High responders had a mean HbA_1c_ of 9.02% (1.36) and low responders had a mean HbA_1c_ of 9.6% (1.51*; P*=.03). High responders and low responders did not differ by race, age, gender, smoking status, diabetes family history, depression or anxiety, or preferred language at baseline.

### Patient SMS Text Messaging Usage and History

At baseline, 73 of 81 (91%) participants indicated they were “very comfortable” or “somewhat comfortable” with SMS text messaging on their phone. In addition, 64 of 81 (79%) participants sent SMS text messages on their phone at least once per day and 68 of 81 (84%) received texts at least once per day. Additionally, 70 of 81 (76%) participants had a smartphone and 74 of 81 (93%) had an unlimited texting plan. A total of 47 of 79 (60%) participants had received SMS text messages from a health care provider or clinic; most had never sent SMS text messages to a health care provider or clinic, used a smartphone app to communicate with a health care provider or clinic, or used a smartphone app to help with their diabetes self-care (Table 1).

### Patient-Reported Outcomes

**Figure 2.**
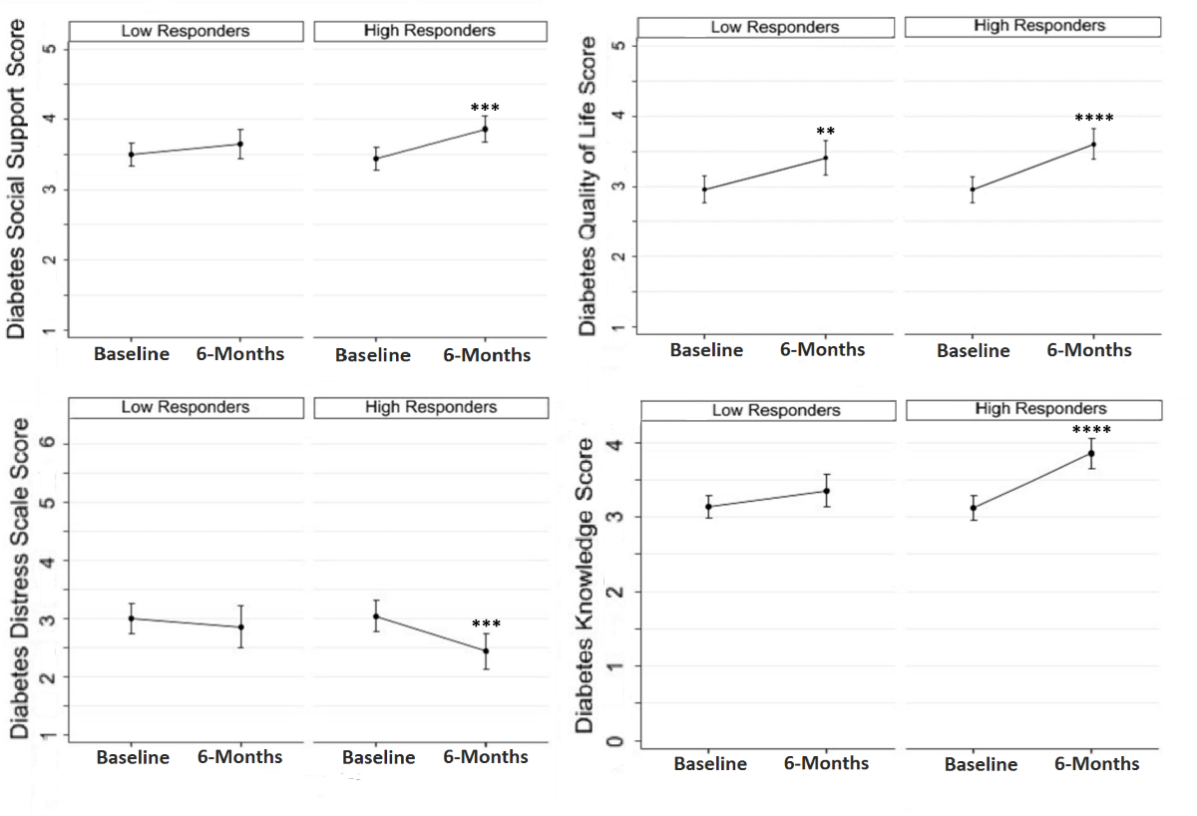
Diabetes social support (upper left, scored 1‐5) [18], diabetes quality of life (upper right, scored 1‐5) [18], diabetes distress (bottom left, scored 1‐6) [17], and diabetes knowledge (bottom right, scored 0‐4) [15] scores from baseline to 6 months in low responders and high responders. Bars represent standard errors.

DDS-2, DQOL, and DSSQ are noted in Figure 2. At baseline, there were no statistically significant differences between high and low responders on DDS-2, DQOL, or DSSQ scores. Within high responders, diabetes distress (*P*=.001), diabetes quality of life (*P*<.001), and diabetes support improved significantly (*P*<.001). Among low responders, diabetes distress (*P*>.05) and diabetes support (*P*>.05) did not improve significantly. Diabetes quality of life did improve significantly among low responders (*P*=.003). Between groups, changes in diabetes distress, diabetes quality of life, and diabetes support between high responders and low responders were not significant.

Diabetes self-care and self-care self-efficacy are noted in Table 2. At baseline, there were no statistically significant differences between high and low responders. Overall, diabetes self-care self-efficacy did not improve significantly for either high (*P*=.06) or low responders (*P*=.94). Changes between groups were also not significant (*P*=.16). Among high responders, diabetes self-care activities improved for foot care (*P*<.001) and general diet practices (*P*=.003). Changes in foot care practices were also significantly different between high and low responders (*P*<.001). For low responders, exercise worsened significantly from baseline to 6 months (*P*=.01) and the change in exercise was significantly different between high and low responder groups (*P*=.002), although high responders did not improve significantly within-group from baseline to 6 months (*P*=.21).

Diabetes care (DKQ) knowledge differences are noted in Figure 2. At baseline, there were no statistically significant differences between high and low responder scores for any DKQ knowledge item. Among high responders, DKQ knowledge improved significantly from baseline to 6 months (*P*<.001). DKQ knowledge did not improve in low responders (*P*=.11). The improvement in DKQ knowledge for high responders was significantly higher than that for low responders in the between-group analysis (*P*<.001).

**Table 2.** Change in glycated hemoglobin A_1c_ and diabetes self-care activities measured by SMS text message engagement status.

	High responder (n=50)				Low responder (n=51)				Between groups at 6 months
	Baseline, mean (SD)	6 months, mean (SD)	*P* value	Total participants	Baseline, mean (SD)	6 months, mean (SD)	*P* value	Total participants	*P* value
Glycated hemoglobin A_1c_ (%)	9.02 (1.36)	8.74 (1.54)	.17	46	9.60 (1.51)	9.22 (1.72)	.27	43	.81
Summary of Diabetes Self-Care Activities measure self-efficacy	3.80 (0.69)	4.08 (0.64)	.06	33	4.08 (0.81)	4.09 (0.79)	.94	23	.16
Foot care	2.64 (2.41)	3.78 (2.43)	<.001	34	3.89 (2.90)	3.37 (2.76)	.28	23	<.001
Exercise	2.30 (1.96)	2.76 (2.08)	.21	34	2.67 (2.56)	2.13 (2.00)	.01	23	.002
Blood sugar testing	4.62 (2.58)	4.72 (2.36)	.68	34	5.46 (1.95)	5.18 (2.34)	.29	23	.50
General diet	3.39 (2.25)	4.21 (1.94)	.003	34	3.86 (2.61)	4.09 (2.41)	.06	23	.69
Specific diet	3.17 (1.67)	3.93 (1.54)	.09	34	4.12 (1.5)	4.00 (1.45)	0.34	23	.87

### Clinical Outcomes

When comparing high responders to low responders at 6 months, there was no significant difference in HbA_1c_. A within-group analysis of high responders and low responders also showed no significant change in HbA_1c_ from baseline to 6 months.

### Satisfaction With the TMI

Overall, 49 of 54 (91%) respondents reported being “very satisfied” (38/54; 70%) or “moderately satisfied” (10/54; 19%) with the SMS text messaging program. Satisfaction was higher among high responders than low responders. Of the high responders, 31 of 34 respondents (91%) reported being “very satisfied” (25/34; 74%) or “moderately satisfied” (6/34; 18%) with the SMS text messaging program. Of the low responders, 15 of 20 respondents (75%) reported being “very satisfied” (10/20; 50%) or “moderately satisfied” (5/20; 25%) with the SMS text messaging program.

Of the 71 patients who answered the free-text questions at the 6-month survey, 23 respondents mentioning they liked the information provided through the SMS text messages. One participant shared that the texts “were very informative with helpful hints and facts about dealing with [their] diabetes.” In addition, 18 mentioned liking logistic functions of the SMS text messaging program, such as the texts acting as reminders and the quiz functions. For example, participants shared that the texts helped with “remembering that [they] need to take care of [themselves]” and that “it was just a nice little reminder a few times a week.” Furthermore, 10 mentioned feeling supported or finding opportunities for reflection through the SMS text messages. Participants shared that the texts “reminded [them] that someone cares,” that they liked “just staying in touch,” that it “[helped them] understand that [they] could do this,” and that the texts “really showed up at times [they] needed a lift.”

When asked what aspects they would change about the SMS text messaging program, 29 of 43 (67%) patients reported that they would change nothing, 6 (14%) wanted more information or content, 5 (12%) wanted the ability to respond to more SMS text messages, and 3 (7%) wanted to change the timing of the messages.

Staff also answered survey free-text questions about the SMS text messaging program and noted both highlights and challenges. Staff noted that the SMS text messaging program was “helpful,” “quick,” and “encouraging” for patients. Several staff also commented on the utility of the SMS text messaging program for patients who may not have been as engaged in GVs:

Although many of our participants didn’t show to every visit, they were still actively participating in the CareMessage program. They liked being able to receive quick, relative information about diabetes in a short text. Many of our patients have very busy lives, so this was an effective way to communicate educational pieces to them.

Patients seemed to be really engaged with the material, even when they weren’t attending the group visits.

Staff also remarked on the challenges of the SMS text messaging program, in particular technological challenges for patients including problems updating patients’ phone numbers that had changed over the course of the program and delivery errors where patients were not getting texts that the program reported were sent. Some staff also wanted more personalization and echoed patients’ wishes regarding being able to respond to additional messages, not only the quizzes:

From my end, there were features I wish it had, like personalized messaging. This way we could have more consistent contact with the participants.

…it would’ve been nicer if patients could’ve responded, a lot of patients wanted to respond but weren’t able to.

## Discussion

### Principal Findings

In this study, we evaluated a 6-month TMI combined with a diabetes GV program in 13 Midwestern FQHC sites serving a diverse population of adults with diabetes. SMS text messaging as a supplement to GV interventions is a novel approach to diabetes interventions. Our study found patients who were more engaged in the SMS text messaging program had significantly higher diabetes care knowledge, had better foot care practices, and exercised more than patients who were less engaged. Highly engaged patients also significantly improved in nearly all patient-reported outcomes including diabetes distress, social support, and quality of life and most self-care practices at 6 months, while low responders did not have any significant changes. Many patients noted feeling supported and encouraged by the SMS text messages. Information was noted as the most helpful aspect of the SMS text messaging program across the cohorts. Unfortunately, these improvements did not translate to clinical improvement in HbA_1c_, unlike what has been seen in prior research. However, the usage of TMIs in vulnerable populations and care sites that serve safety net populations is still emerging. SMS text messaging programs can be a low-burden and effective resource in improving patient-reported outcomes for vulnerable patient populations like those often served by FQHCs, though it may take additional resources to transform improvements in patient-reported outcomes into changes in clinical outcomes.

Though improvements were seen in exercise and foot care, changes in other measured self-care practices were not significantly different between more highly engaged and less engaged patients. These findings may have been due to a high preintervention level of practice or higher barriers to change. Overall, patients already had high frequencies of blood sugar testing at baseline relative to other self-care practices at approximately 5 of 7 days per week and improvements in self-testing were marginal. The improvements seen in foot care may be due to the relatively low barrier of changing foot care behavior compared to other self-care practices. For practices like exercise and diet, behavior change is more resource intensive, requiring patients to have access to and time for healthier eating and exercise, though we did see some improvements. The ability to change diet and exercise practices can be dependent on numerous other factors that cannot be fully addressed by access to information, motivating messages, or self-care reminders.

Unlike considerable prior research, our study did not find significant changes in HbA_1c_ between high and low responders. Generally, systematic analysis and reviews have found that TMIs have a small to moderate effect of reduction of HbA_1c_ in adults with T2DM, though results were variable [4,20,21]. They found that those with a shorter duration of T2DM (<7 years) and a lower HbA_1c_ at baseline had the greatest treatment effects [20]. In our study, the average duration of T2DM was 12.5 years, with an average baseline HbA_1c_ of 9.31%, which may have contributed to the lack of statistically significant improvement in HbA_1c_ despite improvement in patient-reported outcomes. Asian countries and countries of low-to-middle income had greater effects compared to the United States and high-income countries. Other factors evaluated including TMI length, bidirectionality versus unidirectionality, and content and medium of technology were nonsignificant or had conflicting impact among the reviewed analyses [20]. Additional support, resources, and time may be necessary to transform improvements in patient-reported outcomes into improved clinical outcomes, especially in patient populations with long durations of disease and higher baseline HbA_1c_. Several recent studies have similarly not found significant improvements in HbA_1c_ but have found improvements in other outcomes such as patient health engagement and self-empowerment, as well as finding that SMS text messaging programs are generally well-received [7,22].

On average, high responders attended 1 additional GV session than low responders. The difference in GV attendance between high and low responders may be indicative of patient engagement or other willingness or ability to participate in health interventions. To assess for potential confounding between GV attendance and engagement, our models showed that neither GV attendance nor the interaction between GV attendance and SMS text message engagement was significant. Enrollment from the general study population in the TMI was high, as was completion of the program, but survey attrition was also high from baseline to 6 months, especially among low responders. There were few significant differences between high and low responders at baseline. At baseline, there was a statistically significant difference in HbA_1c_ between high and low responders. However, the difference between 9% in high responders and 9.6% in low responders is not a clinically significant difference as both still represent uncontrolled diabetes.

TMIs can provide valuable knowledge for patients by introducing new information and reinforcing previous education. We hypothesized that the TMI served as a continuous source of information that patients received and could access between their diabetes GVs and any individual medical appointments. TMIs can function similarly as being a source of reminders, check-ins, and suggestions for self-care practices in between check-ins with a provider or clinic. Given that many FQHCs can be underresourced and overburdened, TMIs present a largely automated way for patients to receive continuous education, reminders, and suggestions between visits that do not require intensive staff management or clinical appointment time. Although cellphone ownership and SMS text messaging are nearly ubiquitous across socioeconomic statuses, the same cannot be said for smartphone ownership, home broadband access, and comfort with technology. This makes delivery of education and reminders via SMS text messaging (as opposed to smartphone apps or patient portals) extremely important and a catalyst for reducing the digital divide [3]. The TMI was well-received by patients, with both satisfaction and completion being high and patient free-text responses indicating that they liked the content, liked the quizzes, and felt supported. However, both patients and staff had commented on wanting to be able to respond to and engage with additional messages, not only during quizzes. As evident in the literature, it is challenging to discern what features make a TMI and its implementation most effective, though patient satisfaction is usually high [3,20,21]. In an optimized setting, a bidirectional TMI with higher personalization of education and feedback may be most effective and must be considered in the context of lower-resourced clinical settings such as FQHCs [4,20,23].

### Limitations

There are several limitations to this study. The study sites were FQHCs in the Midwest and thus the study may not be generalizable to all FQHC patients**,** though our study population was diverse across many demographics including race, income, and education level. Patient survey responses had considerable attrition at 6-month follow-up, limiting our sample sizes. Patients who were most engaged in care may have been more likely to complete the follow-up survey, thus not representing the total patient sample. Attrition was higher in the online cohort, which is consistent with other research showing survey nonresponse increased during the COVID-19 pandemic [24]. Future studies may consider utilizing additional ways to reach patients for survey completion, such as SMS text messaging or patient portals, to increase response rates. Additionally, due to the COVID-19 pandemic, the delayed cohort ultimately went forward with online GVs, resulting in a different context for their intervention than the original in-person cohort, though the SMS text messaging program remained the same. Analysis comparing the in-person 2018 cohort and the online 2020 cohort was attempted; however, this was limited by the small sample size of the 2020 cohort. Furthermore, there were some technological issues with the implementation of the SMS text messaging program, including having no patients enrolled from 1 site and some patients being unable to receive program texts after changing phone numbers at others.

### Conclusions

This randomized cohort study examined the impact of a TMI combined with a GV intervention in patients with T2DM receiving primary care at FQHCs. Patients with higher TMI engagement had greater improvements in patient-reported outcomes than patients with low engagement; however, clinical improvement was not seen in either group. Further research should examine the part TMIs play in improving patient-reported outcomes and patient clinical outcomes, as well as the relationship between improving them. Additional explorations should investigate what other groups of patients benefit most from TMIs and elucidate which aspects of TMIs best support patients in the FQHC setting. Finally, the integration of TMIs with other health interventions should similarly investigate which patients may benefit the most, what TMI and intervention characteristics are most effective, and how or even if a combined intervention compares to standard approaches.

## Supplementary material

10.2196/55473 Checklist 1 CONSORT E-HEALTH Checklist (V 1.6.1)

## Abbreviations

CHC: community health center
DDS-2: Diabetes Distress Scale
DKQ: Diabetes Knowledge Questionnaire
DQOL: Diabetes Quality of Life
DSSQ: Diabetes Social Support Questionnaire
FQHC: federally qualified health center
GV: group visit
HbA_1c_: glycated hemoglobin A_1c_
MWCN: Midwest Clinicians’ Network
SDSCA: Summary of Diabetes Self-Care Activities
T2DM: type 2 diabetes mellitus
TMI: text messaging intervention
