# Supplementary material for: Impact of a Text Messaging Intervention as an In-Between Support to Diabetes Group Visits in Federally Qualified Health Centers: Cluster Randomized Controlled Study
Source: JMIR Diabetes. 2024 Nov 28;9:e55473. doi: 10.2196/55473 (PMC11619185; doi:10.2196/55473)
Supplement: Checklist 1 [file diabetes-v9-e55473-s001.pdf]

CONSORT-EHEALTH (V 1.6.1) - Submission/Publication Form

The CONSORT-EHEALTH checklist is intended for authors of randomized trials evaluating web-based and Internet-based applications/interventions, including mobile interventions, electronic games (incl multiplayer games), social media, certain telehealth applications, and other interactive and/or networked electronic applications. Some of the items (e.g. all subitems under item 5 - description of the intervention) may also be applicable for other study designs.

The goal of the CONSORT EHEALTH checklist and guideline is to be
a) a guide for reporting for authors of RCTs,
b) to form a basis for appraisal of an ehealth trial (in terms of validity)

CONSORT-EHEALTH items/subitems are MANDATORY reporting items for studies published in the Journal of Medical Internet Research and other journals / scientific societies endorsing the checklist.

Items numbered 1., 2., 3., 4a., 4b etc are original CONSORT or CONSORT-NPT (non-pharmacologic treatment) items.
Items with Roman numerals (i., ii, iii, iv etc.) are CONSORT-EHEALTH extensions/clarifications.

As the CONSORT-EHEALTH checklist is still considered in a formative stage, we would ask that you also RATE ON A SCALE OF 1-5 how important/useful you feel each item is FOR THE PURPOSE OF THE CHECKLIST and reporting guideline (optional).

Mandatory reporting items are marked with a red *.
In the textboxes, either copy & paste the relevant sections from your manuscript into this form - please include any quotes from your manuscript in QUOTATION MARKS,
or answer directly by providing additional information not in the manuscript, or elaborating on why the item was not relevant for this study.

YOUR ANSWERS WILL BE PUBLISHED AS A SUPPLEMENTARY FILE TO YOUR PUBLICATION IN JMIR AND ARE CONSIDERED PART OF YOUR PUBLICATION (IF ACCEPTED).
Please fill in these questions diligently. Information will not be copyedited, so please use proper spelling and grammar, use correct capitalization, and avoid abbreviations.

DO NOT FORGET TO SAVE AS PDF _AND_ CLICK THE SUBMIT BUTTON SO YOUR ANSWERS ARE IN OUR DATABASE !!!

Citation Suggestion (if you append the pdf as Appendix we suggest to cite this paper in the caption):
Eysenbach G, CONSORT-EHEALTH Group
CONSORT-EHEALTH: Improving and Standardizing Evaluation Reports of Web-based and Mobile Health Interventions
J Med Internet Res 2011;13(4):e126
URL: http://www.jmir.org/2011/4/e126/
doi: 10.2196/jmir.1923
PMID: 22209829

alliebot1@gmail.com Switch account

Not shared

Draft saved

* Indicates required question

Your name *

First Last

Arshiya Baig

Primary Affiliation (short), City, Country *

University of Toronto, Toronto, Canada

University of Chicago Medicine, Chicago, USA

Your e-mail address *

abc@gmail.com

abaig@bsd.uchicago.edu

Title of your manuscript *

Provide the (draft) title of your manuscript.

The Impact of Text Messaging Intervention as an In-Between Support to Diabetes Group Visits in Federally Qualified Health Centers: Cluster Randomized Control Study

Name of your App/Software/Intervention *

If there is a short and a long/alternate name, write the short name first and add the long name in brackets.

CareMessage

Evaluated Version (if any)

e.g. "V1", "Release 2017-03-01", "Version 2.0.27913"

Your answer

Language(s) *

What language is the intervention/app in? If multiple languages are available, separate by comma (e.g. "English, French")

English, Spanish

URL of your Intervention Website or App

e.g. a direct link to the mobile app on app in appstore (itunes, Google Play), or URL of the website. If the intervention is a DVD or hardware, you can also link to an Amazon page.

Your answer

URL of an image/screenshot (optional)

Your answer

Accessibility *

Can an enduser access the intervention presently?

- ○ access is free and open
- ○ access only for special usergroups, not open
- ◉ access is open to everyone, but requires payment/subscription/in-app purchases
- ○ app/intervention no longer accessible
- ○ Other:

Primary Medical Indication/Disease/Condition *

e.g. "Stress", "Diabetes", or define the target group in brackets after the condition, e.g. "Autism (Parents of children with)", "Alzheimers (Informal Caregivers of)"

Type 2 Diabetes

Primary Outcomes measured in trial *

comma-separated list of primary outcomes reported in the trial

change in HbA1c, diabetes distress, diabetes k

Secondary/other outcomes

Are there any other outcomes the intervention is expected to affect?

Your answer

Recommended "Dose" *

What do the instructions for users say on how often the app should be used?

- ○ Approximately Daily
- ○ Approximately Weekly
- ○ Approximately Monthly
- ○ Approximately Yearly
- ○ "as needed"
- ● Other: 3-5 times per week

Approx. Percentage of Users (starters) still using the app as recommended after 3 months *

- (x) unknown / not evaluated
- ( ) 0-10%
- ( ) 11-20%
- ( ) 21-30%
- ( ) 31-40%
- ( ) 41-50%
- ( ) 51-60%
- ( ) 61-70%
- ( ) 71%-80%
- ( ) 81-90%
- ( ) 91-100%
- ( ) Other:

Overall, was the app/intervention effective? *

- ( ) yes: all primary outcomes were significantly better in intervention group vs control
- (•) partly: SOME primary outcomes were significantly better in intervention group vs control
- ( ) no statistically significant difference between control and intervention
- ( ) potentially harmful: control was significantly better than intervention in one or more outcomes
- ( ) inconclusive: more research is needed
- ( ) Other:

Article Preparation Status/Stage *

At which stage in your article preparation are you currently (at the time you fill in this form)

- ○ not submitted yet - in early draft status
- ○ not submitted yet - in late draft status, just before submission
- ○ submitted to a journal but not reviewed yet
- ◉ submitted to a journal and after receiving initial reviewer comments
- ○ submitted to a journal and accepted, but not published yet
- ○ published
- ○ Other:

Journal *

If you already know where you will submit this paper (or if it is already submitted), please provide the journal name (if it is not JMIR, provide the journal name under "other")

- ( ) not submitted yet / unclear where I will submit this
- ( ) Journal of Medical Internet Research (JMIR)
- ( ) JMIR mHealth and UHealth
- ( ) JMIR Serious Games
- ( ) JMIR Mental Health
- ( ) JMIR Public Health
- ( ) JMIR Formative Research
- (x) Other JMIR sister journal
- ( ) Other:

Is this a full powered effectiveness trial or a pilot/feasibility trial? *

- ( ) Pilot/feasibility
- (x) Fully powered

Manuscript tracking number *

If this is a JMIR submission, please provide the manuscript tracking number under "other" (The ms tracking number can be found in the submission acknowledgement email, or when you login as author in JMIR. If the paper is already published in JMIR, then the ms tracking number is the four-digit number at the end of the DOI, to be found at the bottom of each published article in JMIR)

- [ ] no ms number (yet) / not (yet) submitted to / published in JMIR
- [x] Other: 55473

TITLE AND ABSTRACT

1a) TITLE: Identification as a randomized trial in the title

1a) Does your paper address CONSORT item 1a? *

I.e does the title contain the phrase "Randomized Controlled Trial"? (if not, explain the reason under "other")

- [x] yes
- [ ] Other:

1a-i) Identify the mode of delivery in the title

Identify the mode of delivery. Preferably use “web-based” and/or “mobile” and/or “electronic game” in the title. Avoid ambiguous terms like “online”, “virtual”, “interactive”. Use “Internet-based” only if Intervention includes non-web-based Internet components (e.g. email), use “computer-based” or “electronic” only if offline products are used. Use “virtual” only in the context of “virtual reality” (3-D worlds). Use “online” only in the context of “online support groups”. Complement or substitute product names with broader terms for the class of products (such as “mobile” or “smart phone” instead of “iphone”), especially if the application runs on different platforms.

	1	2	3	4	5	
subitem not at all important	○	○	○	○	○	essential

Does your paper address subitem 1a-i? *

Copy and paste relevant sections from manuscript title (include quotes in quotation marks "like this" to indicate direct quotes from your manuscript), or elaborate on this item by providing additional information not in the ms, or briefly explain why the item is not applicable/relevant for your study

"The Impact of Text Messaging Intervention as an In-Between Support to Diabetes Group Visits in Federally Qualified Health Centers: Cluster Randomized Control Study"

1a-ii) Non-web-based components or important co-interventions in title

Mention non-web-based components or important co-interventions in title, if any (e.g., “with telephone support”).

	1	2	3	4	5	
subitem not at all important	○	○	○	○	○	essential

Does your paper address subitem 1a-ii?

Copy and paste relevant sections from manuscript title (include quotes in quotation marks "like this" to indicate direct quotes from your manuscript), or elaborate on this item by providing additional information not in the ms, or briefly explain why the item is not applicable/relevant for your study

"The Impact of Text Messaging Intervention as an In-Between Support to Diabetes Group Visits in Federally Qualified Health Centers: Cluster Randomized Control Study"

1a-iii) Primary condition or target group in the title

Mention primary condition or target group in the title, if any (e.g., “for children with Type I Diabetes”) Example: A Web-based and Mobile Intervention with Telephone Support for Children with Type I Diabetes: Randomized Controlled Trial

	1	2	3	4	5	
subitem not at all important	○	○	○	○	○	essential

Does your paper address subitem 1a-iii? *

Copy and paste relevant sections from manuscript title (include quotes in quotation marks "like this" to indicate direct quotes from your manuscript), or elaborate on this item by providing additional information not in the ms, or briefly explain why the item is not applicable/relevant for your study

"The Impact of Text Messaging Intervention as an In-Between Support to Diabetes Group Visits in Federally Qualified Health Centers: Cluster Randomized Control Study"

1b) ABSTRACT: Structured summary of trial design, methods, results, and conclusions

NPT extension: Description of experimental treatment, comparator, care providers, centers, and blinding status.

1b-i) Key features/functionalities/components of the intervention and comparator in the METHODS section of the ABSTRACT

Mention key features/functionalities/components of the intervention and comparator in the abstract. If possible, also mention theories and principles used for designing the site. Keep in mind the needs of systematic reviewers and indexers by including important synonyms. (Note: Only report in the abstract what the main paper is reporting. If this information is missing from the main body of text, consider adding it)

	1	2	3	4	5	
subitem not at all important	○	○	○	○	○	essential

Does your paper address subitem 1b-i? *

Copy and paste relevant sections from the manuscript abstract (include quotes in quotation marks "like this" to indicate direct quotes from your manuscript), or elaborate on this item by providing additional information not in the ms, or briefly explain why the item is not applicable/relevant for your study

"A TMI was implemented for eleven health centers participating in a cluster randomized study of diabetes group visits (GV) in Midwestern FQHCs targeting adults with T2DM. FQHC patients participated in six monthly group visits either in-person or virtually and a concurrent 25-week TMI."

1b-ii) Level of human involvement in the METHODS section of the ABSTRACT

Clarify the level of human involvement in the abstract, e.g., use phrases like “fully automated” vs. “therapist/nurse/care provider/physician-assisted” (mention number and expertise of providers involved, if any). (Note: Only report in the abstract what the main paper is reporting. If this information is missing from the main body of text, consider adding it)

	1	2	3	4	5	
subitem not at all important	○	○	○	○	○	essential

Does your paper address subitem 1b-ii?

Copy and paste relevant sections from the manuscript abstract (include quotes in quotation marks "like this" to indicate direct quotes from your manuscript), or elaborate on this item by providing additional information not in the ms, or briefly explain why the item is not applicable/relevant for your study

CareMessage is automated.

1b-iii) Open vs. closed, web-based (self-assessment) vs. face-to-face assessments in the METHODS section of the ABSTRACT

Mention how participants were recruited (online vs. offline), e.g., from an open access website or from a clinic or a closed online user group (closed usergroup trial), and clarify if this was a purely web-based trial, or there were face-to-face components (as part of the intervention or for assessment). Clearly say if outcomes were self-assessed through questionnaires (as common in web-based trials). Note: In traditional offline trials, an open trial (open-label trial) is a type of clinical trial in which both the researchers and participants know which treatment is being administered. To avoid confusion, use “blinded” or “unblinded” to indicated the level of blinding instead of “open”, as “open” in web-based trials usually refers to “open access” (i.e. participants can self-enrol). (Note: Only report in the abstract what the main paper is reporting. If this information is missing from the main body of text, consider adding it)

	1	2	3	4	5	
subitem not at all important	○	○	○	○	○	essential

Does your paper address subitem 1b-iii?

Copy and paste relevant sections from the manuscript abstract (include quotes in quotation marks "like this" to indicate direct quotes from your manuscript), or elaborate on this item by providing additional information not in the ms, or briefly explain why the item is not applicable/relevant for your study

Health centers were recruited via the Midwest Clinicians' Network (advertised in newsletter, mailed outreach to directors of member health centers), and health centers recruited patients. There were face to face components as this was a text program in combination with a group visit program but the text-messaging component was fully virtual via SMS messaging. Outcomes were assessed via patient completed surveys and analyzing EMR data.

1b-iv) RESULTS section in abstract must contain use data

Report number of participants enrolled/assessed in each group, the use/uptake of the intervention (e.g., attrition/adherence metrics, use over time, number of logins etc.), in addition to primary/secondary outcomes. (Note: Only report in the abstract what the main paper is reporting. If this information is missing from the main body of text, consider adding it)

	1	2	3	4	5	
subitem not at all important	○	○	○	○	○	essential

Does your paper address subitem 1b-iv?

Copy and paste relevant sections from the manuscript abstract (include quotes in quotation marks "like this" to indicate direct quotes from your manuscript), or elaborate on this item by providing additional information not in the ms, or briefly explain why the item is not applicable/relevant for your study

"101 out of 124 GV patients enrolled in the TMI (81.5%). The average age of the population in the TMI was 53 years. Sixty one of 101 respondents (60%) were racial/ethnic minorities, 42 out of 82 respondents (51%) had a high school diploma/GED or less, and 56 out of 80 respondents (71%) reported an annual income <$30,000. Seventy out of 81 respondents (86%) owned a smartphone and 74 out of 80 respondents (93%) had an unlimited texting plan. The median response rate was 41% and the mean response rate was 41.6%. Adjusted models showed significantly improved diabetes knowledge (P < .001), foot care (P < .001), and exercise (P = .002) in high responders (N=34) compared to low responders (N=23) at six months. No group difference was found in HbA1c. Within the high responders group, diabetes distress (P = .001), diabetes social support (P < .001), diabetes quality of life (P < .001), diabetes care knowledge (P < .001), foot care (P < .001), and diet (P = .003) improved from baseline to six months. Low responders only improved in diabetes quality of life (P = .003) from baseline to six months."

1b-v) CONCLUSIONS/DISCUSSION in abstract for negative trials

Conclusions/Discussions in abstract for negative trials: Discuss the primary outcome - if the trial is negative (primary outcome not changed), and the intervention was not used, discuss whether negative results are attributable to lack of uptake and discuss reasons. (Note: Only report in the abstract what the main paper is reporting. If this information is missing from the main body of text, consider adding it)

	1	2	3	4	5	
subitem not at all important	○	○	○	○	○	essential

Does your paper address subitem 1b-v?

Copy and paste relevant sections from the manuscript abstract (include quotes in quotation marks "like this" to indicate direct quotes from your manuscript), or elaborate on this item by providing additional information not in the ms, or briefly explain why the item is not applicable/relevant for your study

Our results section states that "No group difference was found in HbA1c." In our main manuscript body, the conclusion also states this outcome.

INTRODUCTION

2a) In INTRODUCTION: Scientific background and explanation of rationale

2a-i) Problem and the type of system/solution

Describe the problem and the type of system/solution that is object of the study: intended as stand-alone intervention vs. incorporated in broader health care program? Intended for a particular patient population? Goals of the intervention, e.g., being more cost-effective to other interventions, replace or complement other solutions? (Note: Details about the intervention are provided in “Methods” under 5)

	1	2	3	4	5	
subitem not at all important	○	○	○	○	○	essential

Does your paper address subitem 2a-i? *

Copy and paste relevant sections from the manuscript (include quotes in quotation marks "like this" to indicate direct quotes from your manuscript), or elaborate on this item by providing additional information not in the ms, or briefly explain why the item is not applicable/relevant for your study

"One in eleven people receive primary care from a federally qualified health center (FQHC) in the U.S. FQHCs represent a population disproportionately comprised of people with lower socioeconomic status, racial and ethnic minorities, and with a higher disease burden of uncontrolled diabetes. Text-messaging interventions (TMI) have been identified as a promising method to improve clinical outcomes and health behaviors among patients with diabetes... TMIs have been under-studied in vulnerable populations and federally qualified health center (FQHC) patients, which share a disproportionate burden of diabetes."

"However, to date, very few studies have evaluated the usage of TMIs integrated with other clinic- and community-based interventions such as group visits."

"Group visits are a model of care that combines individual medical care, with group education and social support."

"Combining a TMI with a FQHC group visit intervention is a novel approach to addressing diabetes and diabetes disparities. In this study, a combined diabetes group-visit and text messaging intervention was implemented at eleven Midwestern FQHCs with a diverse population of patients with diabetes with suboptimal glycemic control randomized into either a standard in-person cohort or a virtual group visit cohort. The aim of this study was to assess how engagement with a text messaging program correlated with clinical and patient reported outcomes when implemented in the setting of a concurrent group visit program."

2a-ii) Scientific background, rationale: What is known about the (type of) system

Scientific background, rationale: What is known about the (type of) system that is the object of the study (be sure to discuss the use of similar systems for other conditions/diagnoses, if appropiate), motivation for the study, i.e. what are the reasons for and what is the context for this specific study, from which stakeholder viewpoint is the study performed, potential impact of findings [2]. Briefly justify the choice of the comparator.

	1	2	3	4	5	
subitem not at all important	○	○	○	○	○	essential

Does your paper address subitem 2a-ii? *

Copy and paste relevant sections from the manuscript (include quotes in quotation marks "like this" to indicate direct quotes from your manuscript), or elaborate on this item by providing additional information not in the ms, or briefly explain why the item is not applicable/relevant for your study

"Text-messaging interventions (TMI) have been identified as a promising method to improve clinical outcomes and health behaviors among patients with diabetes [2-8]. Many studies have found that TMIs have the potential to improve patient understanding of diabetes, self-efficacy, self-management behaviors, and clinical outcomes such as hemoglobin A1C (HbA1C) among adults with diabetes. TMIs have been under-studied in vulnerable populations and federally qualified health center (FQHC) patients, which share a disproportionate burden of diabetes [3, 6, 9]. A literature review conducted between January 2012 and February 2019 identified only nine original articles studying T2DM text messaging interventions in U.S adults that provided some participant demographics on race, income, or education [9]. Of these articles, only four out of nine reported English-speaking status and two of nine reported income or education [9]. Today, though cellphone ownership is similar across racial groups, education levels, and income levels, disparities still exist with smartphone ownership and home broadband access [10, 11]. With the increasing accessibility and broad use of cell phones and text-messaging amongst vulnerable populations, the barriers to TMIs are continuously decreasing yet research focusing on vulnerable groups remains sparse [9]."

"TMIs are one of the most widely used mobile-health tools and can be used for a wide variety of purposes including medication reminders, provider-patient communication, patient education, motivation, and data collection [2]. However, to date, very few studies have evaluated the usage of TMIs integrated with other clinic- and community-based interventions such as group visits. One prior study combined an interactive and tailored TMI with monthly phone coaching found significant treatment effects on HbA1c at 3-months and 6-months not sustained at later follow-up [12]. Group visits are a model of care that combines individual medical care, with group education and social support. Group visits have been found to be powerful tools in addressing health care inequalities, especially for vulnerable patients with chronic conditions such as diabetes [13, 14]. The application of TMIs in addition to other interventions may be an effective approach to improving outcomes and serve to continue care and contact between patients and providers between visits.

The study of mobile health interventions in vulnerable populations represents both an emerging field of research and opportunity to utilize a powerful tool to serve disadvantaged patient populations that are disproportionately affected by diabetes [6, 9]. Combining a TMI with a FQHC group visit intervention is a novel approach to addressing diabetes and diabetes disparities. In this study, a combined diabetes group-visit and text messaging intervention was implemented at eleven Midwestern FQHCs with a diverse population of [illegible]

patients with diabetes with suboptimal glycemic control randomized into either a standard in-person cohort or a virtual group visit cohort. The aim of this study was to assess how engagement with a text messaging program correlated with clinical and patient reported outcomes when implemented in the setting of a concurrent group visit program."

2b) In INTRODUCTION: Specific objectives or hypotheses

Does your paper address CONSORT subitem 2b? *

Copy and paste relevant sections from the manuscript (include quotes in quotation marks "like this" to indicate direct quotes from your manuscript), or elaborate on this item by providing additional information not in the ms, or briefly explain why the item is not applicable/relevant for your study

"The aim of this study was to assess how engagement with a text messaging program correlated with clinical and patient reported outcomes when implemented in the setting of a concurrent group visit program. "

METHODS

3a) Description of trial design (such as parallel, factorial) including allocation ratio

Does your paper address CONSORT subitem 3a? *

Copy and paste relevant sections from the manuscript (include quotes in quotation marks "like this" to indicate direct quotes from your manuscript), or elaborate on this item by providing additional information not in the ms, or briefly explain why the item is not applicable/relevant for your study

"A cluster randomized controlled study was implemented where FQHC staff conducted a six-month group visit and text messaging intervention at their site or virtually." "Health centers were randomized to either the 2018 in-person intervention arm or the 2020 delayed intervention arm. The 2020 cohort was eventually adapted to a virtual format due to the onset of the COVID-19 pandemic. Sixteen health centers were enrolled. Five withdrew. Six health centers with seven clinical sites were randomized to intervention cohort and five health centers with six clinical sites were randomized to the 2020 delayed intervention. " "FQHC staff invited patients from a randomly ordered list until fifteen patients met the eligibility criteria and agreed to participate. For the virtual 2020 cohort, FQHCs enrolled up to twelve patients due to the virtual format. A total of 124 patients were enrolled in the group visit program. Seventy-five patients were enrolled in the 2018 in person cohort. Forty-nine patients were enrolled in the 2020 virtual cohort." "The in-person FQHCs were asked to implement six monthly 2–3-hour group visits at their care sites based on a previous, successful pilot study. The virtual cohort were asked to implement six monthly 1–2-hour group video visits. Due to the COVID-19 pandemic, the 2020 cohort had video-based virtual group visits." "CareMessage is a nonprofit healthcare patient engagement platform that serves clinical sites with underserved patient populations including FQHCs, free clinics, and other safety-net care settings. It has an automated 25-week interactive Type 2 diabetes SMS based program focusing on self-management and disease education based on national guidelines and supported by medical literature. Of the 124 patients enrolled in the group visit program, 104 patients enrolled in the text messaging program where a total of 101 patients successfully received text messages in the program. "

3b) Important changes to methods after trial commencement (such as eligibility criteria), with reasons

Does your paper address CONSORT subitem 3b? *

Copy and paste relevant sections from the manuscript (include quotes in quotation marks "like this" to indicate direct quotes from your manuscript), or elaborate on this item by providing additional information not in the ms, or briefly explain why the item is not applicable/relevant for your study

"The in-person FQHCs were asked to implement six monthly 2–3-hour group visits at their care sites based on a previous, successful pilot study. The virtual cohort were asked to implement six monthly 1–2-hour group video visits. Due to the COVID-19 pandemic, the 2020 cohort had video-based virtual group visits." The original trial design was for both groups to be in person, however due to the pandemic, the second group was moved to a virtual format.

3b-i) Bug fixes, Downtimes, Content Changes

Bug fixes, Downtimes, Content Changes: ehealth systems are often dynamic systems. A description of changes to methods therefore also includes important changes made on the intervention or comparator during the trial (e.g., major bug fixes or changes in the functionality or content) (5-iii) and other “unexpected events” that may have influenced study design such as staff changes, system failures/downtimes, etc. [2].

	1	2	3	4	5	
subitem not at all important	○	○	○	○	○	essential

Does your paper address subitem 3b-i?

Copy and paste relevant sections from the manuscript (include quotes in quotation marks "like this" to indicate direct quotes from your manuscript), or elaborate on this item by providing additional information not in the ms, or briefly explain why the item is not applicable/relevant for your study

There were no major content changes or bug fixes during the study.

4a) Eligibility criteria for participants

Does your paper address CONSORT subitem 4a? *

Copy and paste relevant sections from the manuscript (include quotes in quotation marks "like this" to indicate direct quotes from your manuscript), or elaborate on this item by providing additional information not in the ms, or briefly explain why the item is not applicable/relevant for your study

"Eligible patients had to be at least 18 years of age, English or Spanish-speaking, have a diagnosis of type 2 diabetes, and a most recently documented glycosylated hemoglobin (A1c) result in the last six months equal to or greater than 8.0%. Patients must have attended at least two appointments at the FQHC within the past year, with at least one of them being from the past six months to ensure that enrolled patients were actively engaged at the FQHC for care. Patients must have owned a cellular phone with text messaging capabilities and have had the ability to send and receive text messages. Patients who were pregnant or planning to become pregnant, had an uncontrolled psychiatric problem, dementia, another cognitive impairment, hearing difficulties, or a severe physical disability that would have excluded them from participation or benefitting from a group visit were excluded."

4a-i) Computer / Internet literacy

Computer / Internet literacy is often an implicit “de facto” eligibility criterion - this should be explicitly clarified.

	1	2	3	4	5	
subitem not at all important	○	○	○	○	○	essential

Does your paper address subitem 4a-i?

Copy and paste relevant sections from the manuscript (include quotes in quotation marks "like this" to indicate direct quotes from your manuscript), or elaborate on this item by providing additional information not in the ms, or briefly explain why the item is not applicable/relevant for your study

"Patients must have owned a cellular phone with text messaging capabilities and have had the ability to send and receive text messages."

4a-ii) Open vs. closed, web-based vs. face-to-face assessments:

Open vs. closed, web-based vs. face-to-face assessments: Mention how participants were recruited (online vs. offline), e.g., from an open access website or from a clinic, and clarify if this was a purely web-based trial, or there were face-to-face components (as part of the intervention or for assessment), i.e., to what degree got the study team to know the participant. In online-only trials, clarify if participants were quasi-anonymous and whether having multiple identities was possible or whether technical or logistical measures (e.g., cookies, email confirmation, phone calls) were used to detect/prevent these.

	1	2	3	4	5	
subitem not at all important	○	○	○	○	○	essential

Does your paper address subitem 4a-ii? *

Copy and paste relevant sections from the manuscript (include quotes in quotation marks "like this" to indicate direct quotes from your manuscript), or elaborate on this item by providing additional information not in the ms, or briefly explain why the item is not applicable/relevant for your study

"The research team sent messages through the Midwest Clinicians' Network (MWCN) listserv, set up a webinar through MCWN, advertised in the quarterly MWCN e-newsletter, and mailed a letter and brochures about the study to the directors of all MWCN member health centers."

"Patients who consented and enrolled in the group visit program were then offered the opportunity to also enroll in the text messaging program which was to run concurrently with the group visit program. Trained CHC staff obtained written informed consent from all intervention participants prior to enrollment. FQHC staff invited patients from a randomly ordered list until fifteen patients met the eligibility criteria and agreed to participate. For the virtual 2020 cohort, FQHCs enrolled up to twelve patients due to the virtual format."

"The in-person FQHCs were asked to implement six monthly 2–3-hour group visits at their care sites based on a previous, successful pilot study. The virtual cohort were asked to implement six monthly 1–2-hour group video visits."

4a-iii) Information giving during recruitment

Information given during recruitment. Specify how participants were briefed for recruitment and in the informed consent procedures (e.g., publish the informed consent documentation as appendix, see also item X26), as this information may have an effect on user self-selection, user expectation and may also bias results.

	1	2	3	4	5	
subitem not at all important	○	○	○	○	○	essential

Does your paper address subitem 4a-iii?

Copy and paste relevant sections from the manuscript (include quotes in quotation marks "like this" to indicate direct quotes from your manuscript), or elaborate on this item by providing additional information not in the ms, or briefly explain why the item is not applicable/relevant for your study

Your answer

4b) Settings and locations where the data were collected

Does your paper address CONSORT subitem 4b? *

Copy and paste relevant sections from the manuscript (include quotes in quotation marks "like this" to indicate direct quotes from your manuscript), or elaborate on this item by providing additional information not in the ms, or briefly explain why the item is not applicable/relevant for your study

"Health centers represented seven Midwestern states including Indiana, Minnesota, Iowa, Wisconsin, Nebraska, Missouri, and Illinois. Specific cities represented both rural and urban settings."

4b-i) Report if outcomes were (self-)assessed through online questionnaires

Clearly report if outcomes were (self-)assessed through online questionnaires (as common in web-based trials) or otherwise.

	1	2	3	4	5	
subitem not at all important	○	○	○	○	○	essential

Does your paper address subitem 4b-i? *

Copy and paste relevant sections from the manuscript (include quotes in quotation marks "like this" to indicate direct quotes from your manuscript), or elaborate on this item by providing additional information not in the ms, or briefly explain why the item is not applicable/relevant for your study

"Patient surveys were administered by health center teams who had multiple options for survey completion including on paper, by phone, or online."

4b-ii) Report how institutional affiliations are displayed

Report how institutional affiliations are displayed to potential participants [on ehealth media], as affiliations with prestigious hospitals or universities may affect volunteer rates, use, and reactions with regards to an intervention.(Not a required item – describe only if this may bias results)

	1	2	3	4	5	
subitem not at all important	○	○	○	○	○	essential

Does your paper address subitem 4b-ii?

Copy and paste relevant sections from the manuscript (include quotes in quotation marks "like this" to indicate direct quotes from your manuscript), or elaborate on this item by providing additional information not in the ms, or briefly explain why the item is not applicable/relevant for your study

Your answer

5) The interventions for each group with sufficient details to allow replication, including how and when they were actually administered

5-i) Mention names, credential, affiliations of the developers, sponsors, and owners

Mention names, credential, affiliations of the developers, sponsors, and owners [6] (if authors/evaluators are owners or developer of the software, this needs to be declared in a “Conflict of interest” section or mentioned elsewhere in the manuscript).

	1	2	3	4	5	
subitem not at all important	○	○	○	○	○	essential

Does your paper address subitem 5-i?

Copy and paste relevant sections from the manuscript (include quotes in quotation marks "like this" to indicate direct quotes from your manuscript), or elaborate on this item by providing additional information not in the ms, or briefly explain why the item is not applicable/relevant for your study

"We’d like to acknowledge the University of Chicago Pritzker School of Medicine as well as the team at CareMessage for their support of this research.
The University of Chicago Institutional Review Board approved all study procedures, and this study was registered at clinicaltrials.gov (NCT03487692) on April 4, 2018. Staff and patients provided written informed consent before participating in the study.
This work was supported by the U.S. Department of Health and Human Services Office of Minority Health (1 CPIMP171145-01-00). The contents of this article are solely the responsibility of the authors and do not necessarily represent the official views of the Office of Minority Health. Additional funding was provided by the Chicago Center for Diabetes Translation Research (NIDDK P30 DK092949) and the University of Chicago Biological Sciences Division Office of Diversity and Inclusion. Study data were collected and managed using REDCap, which is hosted by the University of Chicago Center for Research Informatics and funded by the University of Chicago Biological Sciences Division and the Institute for Translational Medicine (NIH CTSA UL1 TR000430)."

"CareMessage is a nonprofit healthcare patient engagement platform that serves clinical sites with underserved patient populations including FQHCs, free clinics, and other safety-net care settings. It has an automated 25-week interactive Type 2 diabetes SMS based program focusing on self-management and disease education based on national guidelines and supported by medical literature."

5-ii) Describe the history/development process

Describe the history/development process of the application and previous formative evaluations (e.g., focus groups, usability testing), as these will have an impact on adoption/use rates and help with interpreting results.

	1	2	3	4	5	
subitem not at all important	○	○	○	○	○	essential

Does your paper address subitem 5-ii?

Copy and paste relevant sections from the manuscript (include quotes in quotation marks "like this" to indicate direct quotes from your manuscript), or elaborate on this item by providing additional information not in the ms, or briefly explain why the item is not applicable/relevant for your study

Your answer

5-iii) Revisions and updating

Revisions and updating. Clearly mention the date and/or version number of the application/intervention (and comparator, if applicable) evaluated, or describe whether the intervention underwent major changes during the evaluation process, or whether the development and/or content was “frozen” during the trial. Describe dynamic components such as news feeds or changing content which may have an impact on the replicability of the intervention (for unexpected events see item 3b).

	1	2	3	4	5	
subitem not at all important	○	○	○	○	○	essential

Does your paper address subitem 5-iii?

Copy and paste relevant sections from the manuscript (include quotes in quotation marks "like this" to indicate direct quotes from your manuscript), or elaborate on this item by providing additional information not in the ms, or briefly explain why the item is not applicable/relevant for your study

No revisions or updates to the CareMessage program made during intervention periods.

5-iv) Quality assurance methods

Provide information on quality assurance methods to ensure accuracy and quality of information provided [1], if applicable.

	1	2	3	4	5	
subitem not at all important	○	○	○	○	○	essential

Does your paper address subitem 5-iv?

Copy and paste relevant sections from the manuscript (include quotes in quotation marks "like this" to indicate direct quotes from your manuscript), or elaborate on this item by providing additional information not in the ms, or briefly explain why the item is not applicable/relevant for your study

Your answer

5-v) Ensure replicability by publishing the source code, and/or providing screenshots/screen-capture video, and/or providing flowcharts of the algorithms used

Ensure replicability by publishing the source code, and/or providing screenshots/screen-capture video, and/or providing flowcharts of the algorithms used. Replicability (i.e., other researchers should in principle be able to replicate the study) is a hallmark of scientific reporting.

	1	2	3	4	5	
subitem not at all important	○	○	○	○	○	essential

Does your paper address subitem 5-v?

Copy and paste relevant sections from the manuscript (include quotes in quotation marks "like this" to indicate direct quotes from your manuscript), or elaborate on this item by providing additional information not in the ms, or briefly explain why the item is not applicable/relevant for your study

Your answer

5-vi) Digital preservation

Digital preservation: Provide the URL of the application, but as the intervention is likely to change or disappear over the course of the years; also make sure the intervention is archived (Internet Archive, webcitation.org, and/or publishing the source code or screenshots/videos alongside the article). As pages behind login screens cannot be archived, consider creating demo pages which are accessible without login.

	1	2	3	4	5	
subitem not at all important	○	○	○	○	○	essential

Does your paper address subitem 5-vi?

Copy and paste relevant sections from the manuscript (include quotes in quotation marks "like this" to indicate direct quotes from your manuscript), or elaborate on this item by providing additional information not in the ms, or briefly explain why the item is not applicable/relevant for your study

Your answer

5-vii) Access

Access: Describe how participants accessed the application, in what setting/context, if they had to pay (or were paid) or not, whether they had to be a member of specific group. If known, describe how participants obtained “access to the platform and Internet” [1]. To ensure access for editors/reviewers/readers, consider to provide a “backdoor” login account or demo mode for reviewers/readers to explore the application (also important for archiving purposes, see vi).

	1	2	3	4	5	
subitem not at all important	○	○	○	○	○	essential

Does your paper address subitem 5-vii? *

Copy and paste relevant sections from the manuscript (include quotes in quotation marks "like this" to indicate direct quotes from your manuscript), or elaborate on this item by providing additional information not in the ms, or briefly explain why the item is not applicable/relevant for your study

"Patients who consented and enrolled in the group visit program were then offered the opportunity to also enroll in the text messaging program which was to run concurrently with the group visit program."

"Patients must have owned a cellular phone with text messaging capabilities and have had the ability to send and receive text messages."

"CareMessage is a nonprofit healthcare patient engagement platform that serves clinical sites with underserved patient populations including FQHCs, free clinics, and other safety-net care settings. It has an automated 25-week interactive Type 2 diabetes SMS based program focusing on self-management and disease education based on national guidelines and supported by medical literature."

Patients in the study were able to enroll in CareMessage without cost.

5-viii) Mode of delivery, features/functionalities/components of the intervention and comparator, and the theoretical framework

Describe mode of delivery, features/functionalities/components of the intervention and comparator, and the theoretical framework [6] used to design them (instructional strategy [1], behaviour change techniques, persuasive features, etc., see e.g., [7, 8] for terminology). This includes an in-depth description of the content (including where it is coming from and who developed it) [1],” whether [and how] it is tailored to individual circumstances and allows users to track their progress and receive feedback” [6]. This also includes a description of communication delivery channels and – if computer-mediated communication is a component – whether communication was synchronous or asynchronous [6]. It also includes information on presentation strategies [1], including page design principles, average amount of text on pages, presence of hyperlinks to other resources, etc. [1].

	1	2	3	4	5	
subitem not at all important	○	○	○	○	○	essential

Does your paper address subitem 5-viii? *

Copy and paste relevant sections from the manuscript (include quotes in quotation marks "like this" to indicate direct quotes from your manuscript), or elaborate on this item by providing additional information not in the ms, or briefly explain why the item is not applicable/relevant for your study

"CareMessage is a nonprofit healthcare patient engagement platform that serves clinical sites with underserved patient populations including FQHCs, free clinics, and other safety-net care settings. It has an automated 25-week interactive Type 2 diabetes SMS based program focusing on self-management and disease education based on national guidelines and supported by medical literature. Their program contains both unidirectional and bidirectional messages that ask for patient response, including Yes/No, True/False, and multiple-choice questions. Patients received 3-5 texts per week. Messages were available in both English and Spanish, written below a sixth-grade reading level, and culturally tailored for black and Hispanic patients, with content designed to be practical and applicable for low-income patients."

5-ix) Describe use parameters

Describe use parameters (e.g., intended “doses” and optimal timing for use). Clarify what instructions or recommendations were given to the user, e.g., regarding timing, frequency, heaviness of use, if any, or was the intervention used ad libitum.

	1	2	3	4	5	
subitem not at all important	○	○	○	○	○	essential

Does your paper address subitem 5-ix?

Copy and paste relevant sections from the manuscript (include quotes in quotation marks "like this" to indicate direct quotes from your manuscript), or elaborate on this item by providing additional information not in the ms, or briefly explain why the item is not applicable/relevant for your study

"Their program contains both unidirectional and bidirectional messages that ask for patient response, including Yes/No, True/False, and multiple-choice questions. Patients received 3-5 texts per week."

5-x) Clarify the level of human involvement

Clarify the level of human involvement (care providers or health professionals, also technical assistance) in the e-intervention or as co-intervention (detail number and expertise of professionals involved, if any, as well as “type of assistance offered, the timing and frequency of the support, how it is initiated, and the medium by which the assistance is delivered”. It may be necessary to distinguish between the level of human involvement required for the trial, and the level of human involvement required for a routine application outside of a RCT setting (discuss under item 21 – generalizability).

	1	2	3	4	5	
subitem not at all important	○	○	○	○	○	essential

Does your paper address subitem 5-x?

Copy and paste relevant sections from the manuscript (include quotes in quotation marks "like this" to indicate direct quotes from your manuscript), or elaborate on this item by providing additional information not in the ms, or briefly explain why the item is not applicable/relevant for your study

"It has an automated 25-week interactive Type 2 diabetes SMS based program focusing on self-management and disease education based on national guidelines and supported by medical literature."

"CareMessage provided training and worked with the FQHCs to implement the text messaging platform within the clinic's existing infrastructure and workflow."

5-xi) Report any prompts/reminders used

Report any prompts/reminders used: Clarify if there were prompts (letters, emails, phone calls, SMS) to use the application, what triggered them, frequency etc. It may be necessary to distinguish between the level of prompts/reminders required for the trial, and the level of prompts/reminders for a routine application outside of a RCT setting (discuss under item 21 – generalizability).

	1	2	3	4	5	
subitem not at all important	○	○	○	○	○	essential

Does your paper address subitem 5-xi? *

Copy and paste relevant sections from the manuscript (include quotes in quotation marks "like this" to indicate direct quotes from your manuscript), or elaborate on this item by providing additional information not in the ms, or briefly explain why the item is not applicable/relevant for your study

"Their program contains both unidirectional and bidirectional messages that ask for patient response, including Yes/No, True/False, and multiple-choice questions. Patients received 3-5 texts per week."

Patients were enrolled in the program and it was up to them to engage with texts as requested. The texts were automated without a prompt/reminder feature to engage, but would continue to the next text even if the prior one had not been engaged with.

5-xii) Describe any co-interventions (incl. training/support)

Describe any co-interventions (incl. training/support): Clearly state any interventions that are provided in addition to the targeted eHealth intervention, as ehealth intervention may not be designed as stand-alone intervention. This includes training sessions and support [1]. It may be necessary to distinguish between the level of training required for the trial, and the level of training for a routine application outside of a RCT setting (discuss under item 21 – generalizability.

	1	2	3	4	5	
subitem not at all important	○	○	○	○	○	essential

Does your paper address subitem 5-xii? *

Copy and paste relevant sections from the manuscript (include quotes in quotation marks "like this" to indicate direct quotes from your manuscript), or elaborate on this item by providing additional information not in the ms, or briefly explain why the item is not applicable/relevant for your study

"The in-person FQHCs were asked to implement six monthly 2–3-hour group visits at their care sites based on a previous, successful pilot study [13]. The virtual cohort were asked to implement six monthly 1–2-hour group video visits. Due to the COVID-19 pandemic, the 2020 cohort had video-based virtual group visits. The intervention was designed based on a review of the literature and input from staff and providers with experience implementing group visits at health center settings. The group visits contained several core components: (1) an individual medical visit with a provider, (2) group diabetes education, (3) group social support, and (4) goal setting."

6a) Completely defined pre-specified primary and secondary outcome measures, including how and when they were assessed

Does your paper address CONSORT subitem 6a? *

Copy and paste relevant sections from the manuscript (include quotes in quotation marks "like this" to indicate direct quotes from your manuscript), or elaborate on this item by providing additional information not in the ms, or briefly explain why the item is not applicable/relevant for your study

"Patients in both the in-person 2018 cohort and the virtual 2020 cohort completed surveys at baseline and six months, after the last group visit. The patient surveys collected demographic data and data on patients' access, comfort, and prior usage of technology such as texting. Surveys also assessed diabetes knowledge [15] via the Diabetes Knowledge Questionnaire (DKQ), diabetes self-management via the Summary of Diabetes Self-Care Activities scale [16] (SDSCA), SDSCA self-efficacy, diabetes distress [17] (DDS), diabetes-related quality of life [18] (DQOL), and diabetes-related social support [18] (DSS). The diabetes self-care scale (SDSCA) is scored into five sections: foot care, exercise, blood sugar testing, general diet, and specific diet. SDSCA self-efficacy is a survey we developed based on the SDSCA which surveyed patients' confidence in performing those activities. FQHC staff conducted chart reviews to collect patients' clinical outcomes such as HbA1c. Chart data also contained some basic demographic data. Patient surveys also contained free-text portions regarding their experiences with CareMessage.

The CareMessage platform collected data on patient engagement, including the number of texts sent and received, response rates, and retention rates. FQHC teams sent these data reports with deidentified patient information to the University of Chicago team.

Post-intervention, trained research team members from the University of Chicago team conducted 20- to 45-minute telephone interviews with FQHC staff. The interview questions were based on an interview guide designed to assess staff characteristics and involvement; barriers and facilitators to implementing and maintaining a diabetes GVs intervention; characteristics of the group visit intervention as implemented and adapted to their site; desire and ability to sustain the group visit intervention; and perception of patients and staff of the text messaging program. Interviews were audio recorded then transcribed by a professional transcription company."

6a-i) Online questionnaires: describe if they were validated for online use and apply CHERRIES items to describe how the questionnaires were designed/deployed

If outcomes were obtained through online questionnaires, describe if they were validated for online use and apply CHERRIES items to describe how the questionnaires were designed/deployed [9].

	1	2	3	4	5	
subitem not at all important	○	○	○	○	○	essential

Does your paper address subitem 6a-i?

Copy and paste relevant sections from manuscript text

Your answer

6a-ii) Describe whether and how “use” (including intensity of use/dosage) was defined/measured/monitored

Describe whether and how “use” (including intensity of use/dosage) was defined/measured/monitored (logins, logfile analysis, etc.). Use/adoption metrics are important process outcomes that should be reported in any ehealth trial.

	1	2	3	4	5	
subitem not at all important	○	○	○	○	○	essential

Does your paper address subitem 6a-ii?

Copy and paste relevant sections from manuscript text

"The CareMessage platform collected data on patient engagement, including the number of texts sent and received, response rates, and retention rates. FQHC teams sent these data reports with deidentified patient information to the University of Chicago team."

"Response rate was calculated as the number of text messages a patient replied to that were categorized as text messages requesting a response, divided by the total number of text messages that were categorized as ones requiring a response. To grade intervention engagement, patients were classified as high responders if their response rate was equal to or above the median response rate and low responders if their response rate was below the median response rate. Sensitivity analyses were also performed to evaluate response rate as a continuous variable. We defined the median response rate as the median response rate of all patients from both cohorts combined that enrolled in the text messaging program."

6a-iii) Describe whether, how, and when qualitative feedback from participants was obtained

Describe whether, how, and when qualitative feedback from participants was obtained (e.g., through emails, feedback forms, interviews, focus groups).

	1	2	3	4	5	
subitem not at all important	○	○	○	○	○	essential

Does your paper address subitem 6a-iii?

Copy and paste relevant sections from manuscript text

"Post-intervention, trained research team members from the University of Chicago team conducted 20- to 45-minute telephone interviews with FQHC staff. The interview questions were based on an interview guide designed to assess staff characteristics and involvement; barriers and facilitators to implementing and maintaining a diabetes GVs intervention; characteristics of the group visit intervention as implemented and adapted to their site; desire and ability to sustain the group visit intervention; and perception of patients and staff of the text messaging program. Interviews were audio recorded then transcribed by a professional transcription company."

"Patient surveys also contained free-text portions regarding their experiences with CareMessage."

6b) Any changes to trial outcomes after the trial commenced, with reasons

Does your paper address CONSORT subitem 6b? *

Copy and paste relevant sections from the manuscript (include quotes in quotation marks "like this" to indicate direct quotes from your manuscript), or elaborate on this item by providing additional information not in the ms, or briefly explain why the item is not applicable/relevant for your study

There were no changes to outcomes after commencement.

7a) How sample size was determined

NPT: When applicable, details of whether and how the clustering by care provides or centers was addressed

7a-i) Describe whether and how expected attrition was taken into account when calculating the sample size

Describe whether and how expected attrition was taken into account when calculating the sample size.

	1	2	3	4	5	
subitem not at all important	O	O	O	O	O	essential

Does your paper address subitem 7a-i?

Copy and paste relevant sections from manuscript title (include quotes in quotation marks "like this" to indicate direct quotes from your manuscript), or elaborate on this item by providing additional information not in the ms, or briefly explain why the item is not applicable/relevant for your study

In a prior study, power calculations demonstrated that data was needed for 25-35 patients in each arm in order to detect a difference in mean change in A1C between arms of 1.0% +/- 1.0 with a power of 93-98% and a two sided significance level of 5%.

7b) When applicable, explanation of any interim analyses and stopping guidelines

Does your paper address CONSORT subitem 7b? *

Copy and paste relevant sections from the manuscript (include quotes in quotation marks "like this" to indicate direct quotes from your manuscript), or elaborate on this item by providing additional information not in the ms, or briefly explain why the item is not applicable/relevant for your study

There were none applicable.

8a) Method used to generate the random allocation sequence

NPT: When applicable, how care providers were allocated to each trial group

Does your paper address CONSORT subitem 8a? *

Copy and paste relevant sections from the manuscript (include quotes in quotation marks "like this" to indicate direct quotes from your manuscript), or elaborate on this item by providing additional information not in the ms, or briefly explain why the item is not applicable/relevant for your study

Recruited health centers were randomized to intervention or waitlist control in a 1:1 ratio by the study statistician. A block randomization scheme with randomly permuted block sizes of two or four (unknown to the investigation team) was conducted and the randomization was stratified by state and setting (rural versus urban).

8b) Type of randomisation; details of any restriction (such as blocking and block size)

Does your paper address CONSORT subitem 8b? *

Copy and paste relevant sections from the manuscript (include quotes in quotation marks "like this" to indicate direct quotes from your manuscript), or elaborate on this item by providing additional information not in the ms, or briefly explain why the item is not applicable/relevant for your study

This study was cluster randomized.

9) Mechanism used to implement the random allocation sequence (such as sequentially numbered containers), describing any steps taken to conceal the sequence until interventions were assigned

Does your paper address CONSORT subitem 9? *

Copy and paste relevant sections from the manuscript (include quotes in quotation marks "like this" to indicate direct quotes from your manuscript), or elaborate on this item by providing additional information not in the ms, or briefly explain why the item is not applicable/relevant for your study

Recruited health centers were randomized to intervention or waitlist control in a 1:1 ratio by the study statistician. A block randomization scheme with randomly permuted block sizes of two or four (unknown to the investigation team) was conducted and the randomization was stratified by state and setting (rural versus urban).

10) Who generated the random allocation sequence, who enrolled participants, and who assigned participants to interventions

Does your paper address CONSORT subitem 10? *

Copy and paste relevant sections from the manuscript (include quotes in quotation marks "like this" to indicate direct quotes from your manuscript), or elaborate on this item by providing additional information not in the ms, or briefly explain why the item is not applicable/relevant for your study

A biostatistician assigned the health centers.

11a) If done, who was blinded after assignment to interventions (for example, participants, care providers, those assessing outcomes) and how

NPT: Whether or not administering co-interventions were blinded to group assignment

11a-i) Specify who was blinded, and who wasn’t

Specify who was blinded, and who wasn’t. Usually, in web-based trials it is not possible to blind the participants [1, 3] (this should be clearly acknowledged), but it may be possible to blind outcome assessors, those doing data analysis or those administering co-interventions (if any).

	1	2	3	4	5	
subitem not at all important	○	○	○	○	○	essential

Does your paper address subitem 11a-i? *

Copy and paste relevant sections from the manuscript (include quotes in quotation marks "like this" to indicate direct quotes from your manuscript), or elaborate on this item by providing additional information not in the ms, or briefly explain why the item is not applicable/relevant for your study

Blinding was not possible due to the nature of this study.

11a-ii) Discuss e.g., whether participants knew which intervention was the “intervention of interest” and which one was the “comparator”

Informed consent procedures (4a-ii) can create biases and certain expectations - discuss e.g., whether participants knew which intervention was the “intervention of interest” and which one was the “comparator”.

	1	2	3	4	5	
subitem not at all important	○	○	○	○	○	essential

Does your paper address subitem 11a-ii?

Copy and paste relevant sections from the manuscript (include quotes in quotation marks "like this" to indicate direct quotes from your manuscript), or elaborate on this item by providing additional information not in the ms, or briefly explain why the item is not applicable/relevant for your study

Your answer

11b) If relevant, description of the similarity of interventions

(this item is usually not relevant for ehealth trials as it refers to similarity of a placebo or sham intervention to a active medication/intervention)

Does your paper address CONSORT subitem 11b? *

Copy and paste relevant sections from the manuscript (include quotes in quotation marks "like this" to indicate direct quotes from your manuscript), or elaborate on this item by providing additional information not in the ms, or briefly explain why the item is not applicable/relevant for your study

No placebo or sham intervention was used.

12a) Statistical methods used to compare groups for primary and secondary outcomes

NPT: When applicable, details of whether and how the clustering by care providers or centers was addressed

Does your paper address CONSORT subitem 12a? *

Copy and paste relevant sections from the manuscript (include quotes in quotation marks "like this" to indicate direct quotes from your manuscript), or elaborate on this item by providing additional information not in the ms, or briefly explain why the item is not applicable/relevant for your study

"All patient demographics and quantitative outcomes were summarized with descriptive statistics. Linear mixed models (LMM) (for continuous variables) or generalized estimating equations (GEE) (for categorical/binary variables) were used to adjust for within health center association. Each LMM/GEE was also used to examine TMI effects over time by testing effects of TMI, time, and their interaction on each of patient reported outcomes and HbA1c. To consider any potential confounding effects, age, gender, number of group visit attendance, and/or depression or anxiety at baseline were adjusted in these models. Through these models, we also conducted within-group comparison. Furthermore, we examined any cohort effect in each outcome and did not find any evidence to show significance. The interaction term between TMI responder status and group visit attendance in a LMM was not significant and removed from the model. GV attendance was also not significant in the models and thus not included. A two-sided test with $P < 0.05$ was considered statistically significant."

12a-i) Imputation techniques to deal with attrition / missing values

Imputation techniques to deal with attrition / missing values: Not all participants will use the intervention/comparator as intended and attrition is typically high in ehealth trials. Specify how participants who did not use the application or dropped out from the trial were treated in the statistical analysis (a complete case analysis is strongly discouraged, and simple imputation techniques such as LOCF may also be problematic [4]).

	1	2	3	4	5	
subitem not at all important	○	○	○	○	○	essential

Does your paper address subitem 12a-i? *

Copy and paste relevant sections from the manuscript (include quotes in quotation marks "like this" to indicate direct quotes from your manuscript), or elaborate on this item by providing additional information not in the ms, or briefly explain why the item is not applicable/relevant for your study

We did not impute missing values.

12b) Methods for additional analyses, such as subgroup analyses and adjusted analyses

Does your paper address CONSORT subitem 12b? *

Copy and paste relevant sections from the manuscript (include quotes in quotation marks "like this" to indicate direct quotes from your manuscript), or elaborate on this item by providing additional information not in the ms, or briefly explain why the item is not applicable/relevant for your study

"All patient demographics and quantitative outcomes were summarized with descriptive statistics. Linear mixed models (LMM) (for continuous variables) or generalized estimating equations (GEE) (for categorical/binary variables) were used to adjust for within health center association. Each LMM/GEE was also used to examine TMI effects over time by testing effects of TMI, time, and their interaction on each of patient reported outcomes and HbA1c. To consider any potential confounding effects, age, gender, number of group visit attendance, and/or depression or anxiety at baseline were adjusted in these models. Through these models, we also conducted within-group comparison. Furthermore, we examined any cohort effect in each outcome and did not find any evidence to show significance. The interaction term between TMI responder status and group visit attendance in a LMM was not significant and removed from the model. GV attendance was also not significant in the models and thus not included. A two-sided test with P < 0.05 was considered statistically significant."

X26) REB/IRB Approval and Ethical Considerations [recommended as subheading under "Methods"] (not a CONSORT item)

X26-i) Comment on ethics committee approval

	1	2	3	4	5	
subitem not at all important	○	○	○	○	○	essential

Does your paper address subitem X26-i?

Copy and paste relevant sections from the manuscript (include quotes in quotation marks "like this" to indicate direct quotes from your manuscript), or elaborate on this item by providing additional information not in the ms, or briefly explain why the item is not applicable/relevant for your study

"Trained CHC staff obtained written informed consent from all intervention participants prior to enrollment." "The University of Chicago Institutional Review Board approved all study procedures, and this study was registered at clinicaltrials.gov (NCT03487692) on April 4, 2018. Staff and patients provided written informed consent before participating in the study."

x26-ii) Outline informed consent procedures

Outline informed consent procedures e.g., if consent was obtained offline or online (how? Checkbox, etc.?), and what information was provided (see 4a-ii). See [6] for some items to be included in informed consent documents.

	1	2	3	4	5	
subitem not at all important	○	○	○	○	○	essential

Does your paper address subitem X26-ii?

Copy and paste relevant sections from the manuscript (include quotes in quotation marks "like this" to indicate direct quotes from your manuscript), or elaborate on this item by providing additional information not in the ms, or briefly explain why the item is not applicable/relevant for your study

Your answer

X26-iii) Safety and security procedures

Safety and security procedures, incl. privacy considerations, and any steps taken to reduce the likelihood or detection of harm (e.g., education and training, availability of a hotline)

	1	2	3	4	5	
subitem not at all important	○	○	○	○	○	essential

Does your paper address subitem X26-iii?

Copy and paste relevant sections from the manuscript (include quotes in quotation marks "like this" to indicate direct quotes from your manuscript), or elaborate on this item by providing additional information not in the ms, or briefly explain why the item is not applicable/relevant for your study

Your answer

RESULTS

13a) For each group, the numbers of participants who were randomly assigned, received intended treatment, and were analysed for the primary outcome

NPT: The number of care providers or centers performing the intervention in each group and the number of patients treated by each care provider in each center

Does your paper address CONSORT subitem 13a? *

Copy and paste relevant sections from the manuscript (include quotes in quotation marks "like this" to indicate direct quotes from your manuscript), or elaborate on this item by providing additional information not in the ms, or briefly explain why the item is not applicable/relevant for your study

For health centers: "Sixteen health centers were enrolled. Five withdrew. Six health centers with seven clinical sites were randomized to intervention cohort and five health centers with six clinical sites were randomized to the 2020 delayed intervention. Health centers represented seven Midwestern states including Indiana, Minnesota, Iowa, Wisconsin, Nebraska, Missouri, and Illinois. Specific cities represented both rural and urban settings."

Figure 1 shows the patient numbers from the in-person cohort and the virtual cohort that were consented and enrolled in the text messaging program and subsequently completed the surveys. "A total of 124 patients were enrolled in the group visit program. Seventy-five patients were enrolled in the 2018 in person cohort. Forty-nine patients were enrolled in the 2020 virtual cohort." "Of the 124 patients enrolled in the group visit program, 104 patients enrolled in the text messaging program where a total of 101 patients successfully received text messages in the program. Full enrollment data is presented in Figure 1."

13b) For each group, losses and exclusions after randomisation, together with reasons

Does your paper address CONSORT subitem 13b? (NOTE: Preferably, this is shown in a CONSORT flow diagram) *

Copy and paste relevant sections from the manuscript (include quotes in quotation marks "like this" to indicate direct quotes from your manuscript), or elaborate on this item by providing additional information not in the ms, or briefly explain why the item is not applicable/relevant for your study

An enrollment and survey completion diagram is included in this manuscript.

13b-i) Attrition diagram

Strongly recommended: An attrition diagram (e.g., proportion of participants still logging in or using the intervention/comparator in each group plotted over time, similar to a survival curve) or other figures or tables demonstrating usage/dose/engagement.

	1	2	3	4	5	
subitem not at all important	○	○	○	○	○	essential

Does your paper address subitem 13b-i?

Copy and paste relevant sections from the manuscript or cite the figure number if applicable (include quotes in quotation marks "like this" to indicate direct quotes from your manuscript), or elaborate on this item by providing additional information not in the ms, or briefly explain why the item is not applicable/relevant for your study

Your answer

14a) Dates defining the periods of recruitment and follow-up

Does your paper address CONSORT subitem 14a? *

Copy and paste relevant sections from the manuscript (include quotes in quotation marks "like this" to indicate direct quotes from your manuscript), or elaborate on this item by providing additional information not in the ms, or briefly explain why the item is not applicable/relevant for your study

The 2018 cohort recruitment period was from October of 2018 until January of 2019. The intervention occurred from April of 2019 until July of 2019. Data collection was completed by September of 2019.

The 2020 cohort recruitment period was from September of 2020 until March of 2021. The intervention occurred from March of 2021 until August of 2021. Data collection was completed by September of 2021.

14a-i) Indicate if critical "secular events" fell into the study period

Indicate if critical "secular events" fell into the study period, e.g., significant changes in Internet resources available or "changes in computer hardware or Internet delivery resources"

	1	2	3	4	5	
subitem not at all important	○	○	○	○	○	essential

Does your paper address subitem 14a-i?

Copy and paste relevant sections from the manuscript (include quotes in quotation marks "like this" to indicate direct quotes from your manuscript), or elaborate on this item by providing additional information not in the ms, or briefly explain why the item is not applicable/relevant for your study

Your answer

14b) Why the trial ended or was stopped (early)

Does your paper address CONSORT subitem 14b? *

Copy and paste relevant sections from the manuscript (include quotes in quotation marks "like this" to indicate direct quotes from your manuscript), or elaborate on this item by providing additional information not in the ms, or briefly explain why the item is not applicable/relevant for your study

The trial was not stopped prematurely.

15) A table showing baseline demographic and clinical characteristics for each group

NPT: When applicable, a description of care providers (case volume, qualification, expertise, etc.) and centers (volume) in each group

Does your paper address CONSORT subitem 15? *

Copy and paste relevant sections from the manuscript (include quotes in quotation marks "like this" to indicate direct quotes from your manuscript), or elaborate on this item by providing additional information not in the ms, or briefly explain why the item is not applicable/relevant for your study

There is a baseline demographic and clinical characteristic table included in the [illegible]

15-i) Report demographics associated with digital divide issues

In ehealth trials it is particularly important to report demographics associated with digital divide issues, such as age, education, gender, social-economic status, computer/Internet/ehealth literacy of the participants, if known.

	1	2	3	4	5	
subitem not at all important	○	○	○	○	○	essential

Does your paper address subitem 15-i? *

Copy and paste relevant sections from the manuscript (include quotes in quotation marks "like this" to indicate direct quotes from your manuscript), or elaborate on this item by providing additional information not in the ms, or briefly explain why the item is not applicable/relevant for your study

Characteristics associated with digital divide issues are reported in the paper including age, education, gender, income, and phone ownership and usage comfort in the demographics table.

16) For each group, number of participants (denominator) included in each analysis and whether the analysis was by original assigned groups

16-i) Report multiple "denominators" and provide definitions

Report multiple "denominators" and provide definitions: Report N's (and effect sizes) "across a range of study participation [and use] thresholds" [1], e.g., N exposed, N consented, N used more than x times, N used more than y weeks, N participants "used" the intervention/comparator at specific pre-defined time points of interest (in absolute and relative numbers per group). Always clearly define "use" of the intervention.

	1	2	3	4	5	
subitem not at all important	○	○	○	○	○	essential

Does your paper address subitem 16-i? *

Copy and paste relevant sections from the manuscript (include quotes in quotation marks "like this" to indicate direct quotes from your manuscript), or elaborate on this item by providing additional information not in the ms, or briefly explain why the item is not applicable/relevant for your study

"Table 1 describes the demographics and baseline characteristics of patients who enrolled in the text messaging program (N=101)." Each specific item has its own sample size in the table due to survey completion discrepancies.

16-ii) Primary analysis should be intent-to-treat

Primary analysis should be intent-to-treat, secondary analyses could include comparing only “users”, with the appropriate caveats that this is no longer a randomized sample (see 18-i).

	1	2	3	4	5	
subitem not at all important	O	O	O	O	O	essential

Does your paper address subitem 16-ii?

Copy and paste relevant sections from the manuscript (include quotes in quotation marks "like this" to indicate direct quotes from your manuscript), or elaborate on this item by providing additional information not in the ms, or briefly explain why the item is not applicable/relevant for your study

The primary analysis was intention to treat.

17a) For each primary and secondary outcome, results for each group, and the estimated effect size and its precision (such as 95% confidence interval)

Does your paper address CONSORT subitem 17a? *

Copy and paste relevant sections from the manuscript (include quotes in quotation marks "like this" to indicate direct quotes from your manuscript), or elaborate on this item by providing additional information not in the ms, or briefly explain why the item is not applicable/relevant for your study

Effect size is shown in the tables and figures in the results section. P-values are reported according to the journal guidelines: exact values are used as appropriate unless less than .001. Standard errors are shown in Figure 2.

17a-i) Presentation of process outcomes such as metrics of use and intensity of use

In addition to primary/secondary (clinical) outcomes, the presentation of process outcomes such as metrics of use and intensity of use (dose, exposure) and their operational definitions is critical. This does not only refer to metrics of attrition (13-b) (often a binary variable), but also to more continuous exposure metrics such as “average session length”. These must be accompanied by a technical description how a metric like a “session” is defined (e.g., timeout after idle time) [1] (report under item 6a).

	1	2	3	4	5	
subitem not at all important	○	○	○	○	○	essential

Does your paper address subitem 17a-i?

Copy and paste relevant sections from the manuscript (include quotes in quotation marks "like this" to indicate direct quotes from your manuscript), or elaborate on this item by providing additional information not in the ms, or briefly explain why the item is not applicable/relevant for your study

Responder status and response rate definitions are defined in the manuscript to represent patient usage of the TMI.

17b) For binary outcomes, presentation of both absolute and relative effect sizes is recommended

Does your paper address CONSORT subitem 17b? *

Copy and paste relevant sections from the manuscript (include quotes in quotation marks "like this" to indicate direct quotes from your manuscript), or elaborate on this item by providing additional information not in the ms, or briefly explain why the item is not applicable/relevant for your study

Outcomes were not binary. Absolute survey scores and values are shown in a table and figure included in the submission.

18) Results of any other analyses performed, including subgroup analyses and adjusted analyses, distinguishing pre-specified from exploratory

Does your paper address CONSORT subitem 18? *

Copy and paste relevant sections from the manuscript (include quotes in quotation marks "like this" to indicate direct quotes from your manuscript), or elaborate on this item by providing additional information not in the ms, or briefly explain why the item is not applicable/relevant for your study

All results of planned analyses are shown. No additional analyses done after the fact.

18-i) Subgroup analysis of comparing only users

A subgroup analysis of comparing only users is not uncommon in ehealth trials, but if done, it must be stressed that this is a self-selected sample and no longer an unbiased sample from a randomized trial (see 16-iii).

	1	2	3	4	5	
subitem not at all important	○	○	○	○	○	essential

Does your paper address subitem 18-i?

Copy and paste relevant sections from the manuscript (include quotes in quotation marks "like this" to indicate direct quotes from your manuscript), or elaborate on this item by providing additional information not in the ms, or briefly explain why the item is not applicable/relevant for your study

Your answer

19) All important harms or unintended effects in each group

(for specific guidance see CONSORT for harms)

Does your paper address CONSORT subitem 19? *

Copy and paste relevant sections from the manuscript (include quotes in quotation marks "like this" to indicate direct quotes from your manuscript), or elaborate on this item by providing additional information not in the ms, or briefly explain why the item is not applicable/relevant for your study

We did not find any harms or unintended effects in each group.

19-i) Include privacy breaches, technical problems

Include privacy breaches, technical problems. This does not only include physical "harm" to participants, but also incidents such as perceived or real privacy breaches [1], technical problems, and other unexpected/unintended incidents. "Unintended effects" also includes unintended positive effects [2].

	1	2	3	4	5	
subitem not at all important	○	○	○	○	○	essential

Does your paper address subitem 19-i?

Copy and paste relevant sections from the manuscript (include quotes in quotation marks "like this" to indicate direct quotes from your manuscript), or elaborate on this item by providing additional information not in the ms, or briefly explain why the item is not applicable/relevant for your study

There were no privacy breaches in this study. Technical problems were reported.

19-ii) Include qualitative feedback from participants or observations from staff/researchers

Include qualitative feedback from participants or observations from staff/researchers, if available, on strengths and shortcomings of the application, especially if they point to unintended/unexpected effects or uses. This includes (if available) reasons for why people did or did not use the application as intended by the developers.

	1	2	3	4	5	
subitem not at all important	○	○	○	○	○	essential

Does your paper address subitem 19-ii?

Copy and paste relevant sections from the manuscript (include quotes in quotation marks "like this" to indicate direct quotes from your manuscript), or elaborate on this item by providing additional information not in the ms, or briefly explain why the item is not applicable/relevant for your study

Qualitative outcomes was included in the manuscript results section.

DISCUSSION

22) Interpretation consistent with results, balancing benefits and harms, and considering other relevant evidence

NPT: In addition, take into account the choice of the comparator, lack of or partial blinding, and unequal expertise of care providers or centers in each group

22-i) Restate study questions and summarize the answers suggested by the data, starting with primary outcomes and process outcomes (use)

Restate study questions and summarize the answers suggested by the data, starting with primary outcomes and process outcomes (use).

	1	2	3	4	5	
subitem not at all important	○	○	○	○	○	essential

Does your paper address subitem 22-i? *

Copy and paste relevant sections from the manuscript (include quotes in quotation marks "like this" to indicate direct quotes from your manuscript), or elaborate on this item by providing additional information not in the ms, or briefly explain why the item is not applicable/relevant for your study

"In this study, we evaluated a 6-month text messaging intervention combined with a diabetes group visit program in thirteen Midwestern FQHC sites serving a diverse population of adults with diabetes. Text messaging as a supplement to group visit interventions is a novel approach to diabetes interventions. Our study found patients who were more engaged in the text messaging program had significantly higher diabetes care knowledge, foot care practices, and exercise than patients who were less engaged. Highly engaged patients also significantly improved in nearly all patient reported outcomes including diabetes distress, social support, and quality of life and most self-care practices at six months while low responders did not have any significant changes. Many patients noted feeling supported and encouraged by the text messages. Information was noted as the most helpful aspect of the text messaging program across the cohorts. Unfortunately, these improvements did not translate to clinical improvement in A1c unlike what has been seen in prior research."

22-ii) Highlight unanswered new questions, suggest future research

Highlight unanswered new questions, suggest future research.

	1	2	3	4	5	
subitem not at all important	○	○	○	○	○	essential

Does your paper address subitem 22-ii?

Copy and paste relevant sections from the manuscript (include quotes in quotation marks "like this" to indicate direct quotes from your manuscript), or elaborate on this item by providing additional information not in the ms, or briefly explain why the item is not applicable/relevant for your study

"Further research should examine the part TMIs play in improving patient reported outcomes and patient clinical outcomes as well as the relationship between improving them. Additional explorations should investigate what other groups of patients benefit most from TMIs and elucidate what aspects of TMIs best support patients in the FQHC setting. Lastly, the integration of TMIs with other health interventions should similarly be studied for which patients may benefit the most, what TMI and intervention characteristics are most effective, and how or even if a combined intervention compare to standard approaches."

20) Trial limitations, addressing sources of potential bias, imprecision, and, if relevant, multiplicity of analyses

20-i) Typical limitations in ehealth trials

Typical limitations in ehealth trials: Participants in ehealth trials are rarely blinded. Ehealth trials often look at a multiplicity of outcomes, increasing risk for a Type I error. Discuss biases due to non-use of the intervention/usability issues, biases through informed consent procedures, unexpected events.

	1	2	3	4	5	
subitem not at all important	○	○	○	○	○	essential

Does your paper address subitem 20-i? *

Copy and paste relevant sections from the manuscript (include quotes in quotation marks "like this" to indicate direct quotes from your manuscript), or elaborate on this item by providing additional information not in the ms, or briefly explain why the item is not applicable/relevant for your study

"Patient survey responses had considerable attrition at six-month follow-up, limiting our end sample sizes. It is possible that due to patients lost to follow-up, there was not enough power to see differences in A1c between the two groups. Selection bias may also influence results. Though patients were recruited from randomly ordered lists, they had to first consent to group visit interventions and then consent to the text messaging program. This may have resulted in a patient population that was more motivated to buy-in than the general population. Furthermore, many of our outcomes utilized patient-reporting which can be prone to bias."

21) Generalisability (external validity, applicability) of the trial findings

NPT: External validity of the trial findings according to the intervention, comparators, patients, and care providers or centers involved in the trial

21-i) Generalizability to other populations

Generalizability to other populations: In particular, discuss generalizability to a general Internet population, outside of a RCT setting, and general patient population, including applicability of the study results for other organizations

	1	2	3	4	5	
subitem not at all important	○	○	○	○	○	essential

Does your paper address subitem 21-i?

Copy and paste relevant sections from the manuscript (include quotes in quotation marks "like this" to indicate direct quotes from your manuscript), or elaborate on this item by providing additional information not in the ms, or briefly explain why the item is not applicable/relevant for your study

"The study sites were FQHCs in the Midwest and thus may not be generalizable to all FQHC patients, though our study population was diverse across many demographics including race, income, and education level."

21-ii) Discuss if there were elements in the RCT that would be different in a routine application setting

Discuss if there were elements in the RCT that would be different in a routine application setting (e.g., prompts/reminders, more human involvement, training sessions or other co-interventions) and what impact the omission of these elements could have on use, adoption, or outcomes if the intervention is applied outside of a RCT setting.

	1	2	3	4	5	
subitem not at all important	○	○	○	○	○	essential

Does your paper address subitem 21-ii?

Copy and paste relevant sections from the manuscript (include quotes in quotation marks "like this" to indicate direct quotes from your manuscript), or elaborate on this item by providing additional information not in the ms, or briefly explain why the item is not applicable/relevant for your study

Your answer

OTHER INFORMATION

23) Registration number and name of trial registry

Does your paper address CONSORT subitem 23? *

Copy and paste relevant sections from the manuscript (include quotes in quotation marks "like this" to indicate direct quotes from your manuscript), or elaborate on this item by providing additional information not in the ms, or briefly explain why the item is not applicable/relevant for your study

"Trial Registration: Clincialtrials.gov NCT03487692, https://clinicaltrials.gov/study/NCT03487692"

24) Where the full trial protocol can be accessed, if available

Does your paper address CONSORT subitem 24? *

Cite a Multimedia Appendix, other reference, or copy and paste relevant sections from the manuscript (include quotes in quotation marks "like this" to indicate direct quotes from your manuscript), or elaborate on this item by providing additional information not in the ms, or briefly explain why the item is not applicable/relevant for your study

The full trial protocol can be requested from the corresponding author.

25) Sources of funding and other support (such as supply of drugs), role of funders

Does your paper address CONSORT subitem 25? *

Copy and paste relevant sections from the manuscript (include quotes in quotation marks "like this" to indicate direct quotes from your manuscript), or elaborate on this item by providing additional information not in the ms, or briefly explain why the item is not applicable/relevant for your study

"We'd like to acknowledge the University of Chicago Pritzker School of Medicine as well as the team at CareMessage for their support of this research.
The University of Chicago Institutional Review Board approved all study procedures, and this study was registered at clinicaltrials.gov (NCT03487692) on April 4, 2018. Staff and patients provided written informed consent before participating in the study.
This work was supported by the U.S. Department of Health and Human Services Office of Minority Health (1 CPIMP171145-01-00). The contents of this article are solely the responsibility of the authors and do not necessarily represent the official views of the Office of Minority Health. Additional funding was provided by the Chicago Center for Diabetes Translation Research (NIDDK P30 DK092949) and the University of Chicago Biological Sciences Division Office of Diversity and Inclusion. Study data were collected and managed using REDCap, which is hosted by the University of Chicago Center for Research Informatics and funded by the University of Chicago Biological Sciences Division and the Institute for Translational Medicine (NIH CTSA UL1 TR000430)."

X27) Conflicts of Interest (not a CONSORT item)

X27-i) State the relation of the study team towards the system being evaluated

In addition to the usual declaration of interests (financial or otherwise), also state the relation of the study team towards the system being evaluated, i.e., state if the authors/evaluators are distinct from or identical with the developers/sponsors of the intervention.

	1	2	3	4	5	
subitem not at all important	○	○	○	○	○	essential

Does your paper address subitem X27-i?

Copy and paste relevant sections from the manuscript (include quotes in quotation marks "like this" to indicate direct quotes from your manuscript), or elaborate on this item by providing additional information not in the ms, or briefly explain why the item is not applicable/relevant for your study

"The authors have no conflicts of interest to disclose."

About the CONSORT EHEALTH checklist

As a result of using this checklist, did you make changes in your manuscript? *

- ○ yes, major changes
- ◉ yes, minor changes
- ○ no

What were the most important changes you made as a result of using this checklist?

Your answer

How much time did you spend on going through the checklist INCLUDING making changes in your manuscript *

Approximately seven hours.

As a result of using this checklist, do you think your manuscript has improved? *

- [x] yes
- [ ] no
- [ ] Other:

Would you like to become involved in the CONSORT EHEALTH group?

This would involve for example becoming involved in participating in a workshop and writing an "Explanation and Elaboration" document

- [ ] yes
- [ ] no
- [ ] Other:

Any other comments or questions on CONSORT EHEALTH

Your answer

STOP - Save this form as PDF before you click submit

To generate a record that you filled in this form, we recommend to generate a PDF of this page (on a Mac, simply select "print" and then select "print as PDF") before you submit it.

When you submit your (revised) paper to JMIR, please upload the PDF as supplementary file.

Don't worry if some text in the textboxes is cut off, as we still have the complete information in our database. Thank you!

Final step: Click submit !

Click submit so we have your answers in our database!

Submit   Clear form

Never submit passwords through Google Forms.

This content is neither created nor endorsed by Google. Report Abuse - Terms of Service - Privacy Policy

Google Forms
